# Environmental and sustainable valorization of spent adsorbent: safety and acute toxicity evaluation in rats via probit analysis

**DOI:** 10.1038/s41598-026-50808-4

**Published:** 2026-05-18

**Authors:** Saher A. Aita, Rehab Mahmoud, Fatma I. Abo El-Ela, Esraa G. Arafa, Amal Zaher

**Affiliations:** 1 Environmental Science and Industrial Development Department, Faculty of Postgraduate Studies for Advanced Sciences, Beni-Suef University, Beni-Suef, 62511 Egypt; 2 Chemistry Department, Faculty of Science, Beni-Suef University, Beni-Suef, 62511 Egypt; 3 Department of Pharmacology, Faculty of Veterinary Medicine, Beni-Suef University, Beni-Suef, 62511 Egypt

**Keywords:** Biochemical parameters, Hematological analysis, Toxicity, Safety, Adsorbent spent, LDH, Biochemistry, Biological techniques, Environmental sciences

## Abstract

Valorization of adsorbent spent is a modern trend to maximize the utilization of absorption waste. Analytical methods, inclusive Fourier Transform Infra Red (FTIR) spectroscopy, Scanning Electron Microscope (SEM), and X-Ray Diffraction (XRD), were applied to evaluate the adsorbent spent (Zn-Co-Fe/LDH @ heavy metal). We examined the mechanistic toxicological effects of heavy metals within an animal model (rats) via probit analysis to evaluate the safety and acute toxicity of the spent adsorbent. The acute toxicity was evaluated and documented for 24 h, and it persisted for 14 days. A diverse multitude of toxic impacts on a different of body organs and tissues is the consequence of the bioaccumulation of these heavy metals. The rodents were weighed daily, and a variety of observations, like mortality, injury, behavior, and any indications of disease, were conducted. The safety and toxicological analyses were conducted using Probit analysis. The acute toxicity evaluation revealed varying safety profiles for the spent adsorbents. Zn-Co-Fe/LDH-As exhibited the lowest level of toxicity with an LD_50_ of 370 mg/kg and a calculated safe dose of 18.5 mg/kg. In contrast, the highest toxicity was observed for Zn-Co-Fe/LDH-Pb (LD_50_ = 103.7 mg/kg; safe dose = 5.2 mg/kg), followed by Zn-Co-Fe/LDH-Hg (LD_50_ = 204 mg/kg; safe dose = 10.2 mg/kg). These results quantify the biological risk associated with metal-laden adsorbents and establish the safety benchmarks required for their sustainable valorization. Evaluations of biochemical parameters and hematological analysis were conducted at the conclusion of each investigation. Comparative to the controls, gross findings were obtained from the histopathological examination of the animals’ vital organs, which included the heart, lung, kidney, liver, and stomach. Our safety and toxicological data demonstrated the safety of layered double hydroxide (LDH) efficacy in adsorption, as well as a lower level of toxicity for LDH/As after adsorption. The iron content within the Zn-Co-Fe/LDH framework remained structurally integrated; however, the observed hematological alterations suggest a potential interference with iron metabolism or heme synthesis following exposure to heavy-metal laden adsorbents.

**Supplementary Information:**

The online version contains supplementary material available at 10.1038/s41598-026-50808-4.

## Introduction

Exposure to heavy metals has grown due to modern industrialization and anthropogenic processes, which have negative effects on human health. The pollution of air and water with toxic metal is a significant environmental issue impacting hundreds of millions of people. Both animal and human health is also concerned about food contamination with heavy metals. The quantity of heavy metals present in food, air, and water is monitored^1–4^. The metallic elements known as heavy metals, which include lead, arsenic, chromium, aluminum and mercury can cause toxicity for humans and other vertebrates at extremely low concentrations (Mubarak et at., 2020). They do this through interfering with cellular organelles like cell membranes and, mitochondrial lysosomes which disrupts metabolism, tissue repair, and cell and organ detoxification^5^, Heavy metals exert toxicity by interfering with critical cellular structures, including the endothelial glycocalyx, cell membranes, and mitochondrial lysosomes. This interference disrupts essential processes such as metabolism, tissue repair, and the mechanisms of cell and organ detoxification^6^. Additionally, they have the ability to assault and react with DNA molecules to encourage carcinogenesis, apoptosis, and cell cycle modification (Reyersmann et at., 2008).

One of the major heavy metals that pose a health danger to the population is arsenic. Exposure to As can occur from the workplace or via tainted water and food. It has an extended history of usage, both as a medical medication and as a metalloid material. It is infamously referred to as the poison of kings and the king of poisons^7^. As is a pollutant that may be present in the environment, and water food. There are four different types of arsenic: arsine (AsH3), inorganic (As^3+^ and As^5+^), metalloid (As0), and organic. The order of increasing toxicity of As compounds is defined as organic arsenicals < As0 < Inorganic species (As^5+^  < As^3+)^ < arsine^8–11^. In industrial wastewater, concentrations of arsenic (As), lead (Pb), and mercury (Hg) frequently exceed the permissible discharge limits set by regulatory bodies like the WHO and EPA. While these metals are traditionally studied in their ionic forms, their presence in spent adsorbents poses a unique health risk during handling and disposal. For instance, Pb concentrations in battery and smelting effluents can reach levels that induce acute neurotoxicity and renal failure in mammalian models. This study directly addresses the safety concerns associated with the high-load sequestration of these toxins from industrial streams into solid waste matrices^4^.

Lead is a dangerous environmental contaminant that is extremely hazardous to various bodily organs. Pb is mostly absorbed through the digestive and respiratory systems, despite the fact that it can be absorbed through the skin. Pb exposure can cause oxidative stress, inflammation, immunological modulation, and respiratory, pulmonary, and cardiovascular diseases. Moreover, lead (Pb) may throw off the equilibrium of the antioxidant-oxidant system and cause inflammatory reactions in different organs. Exposure to lead (Pb) has been linked to numerous ailments and can change the body’s physiological systems^12–14^. Exposure to lead triggers the production of B and T-cells as well as MHC activity^15^. It can influence cellular and humoral responses by modifying the role of T-cell and increasing susceptibility to development of autoimmunity and hypersensitivity^16,17^. Lead is so toxic, it negatively impacts the body’s neurological, biochemical, and cognitive systems. The blood level of concern for lead poisoning worldwide is 10 μg/dl^18,19^. In recent years, adulteration of opium with lead has been viewed as a health risk to humans^20^.

Organic mercury compounds are thought to be the primary source of mercury entering the aquatic environment, with concentrations often not exceeding 0.1 mg/L^21^. Mercury is commonly employed as a fungicide in farms. One of the most harmful substances to fish is methyl mercury, which is typically produced by anaerobic microorganisms such as iron reducers, sulfate-reducing bacteria, and methanogens MPA methylating inorganic mercury^22^. Methyl mercury is a very soluble environmental pollutant that was initially identified in 1970 as the source of widespread people poisoning in Iraq and contamination in Japan’s Minamata Bay^23^^,^^24^.

LDHs are a promising and widespread class of two-dimensional (2D) inorganic layered nanomaterials, known as synthetic or natural anionic hydrotalcite (Mg_6_Al_2_(OH)_16_CO_3_·4H_2_O)-like clays, with positive and negative charges of solvent molecules between their interlayers. Among the natural forms of LDHs, hydrotalcite, as a member of the LDH family, was first discovered in 1842, and named so because of its high water (that is, hydro) and resemblance to talc^25^.

Materials known as layered double hydroxides (LDHs) are composed of brucite-like layers in which six hydroxyl groups coordinate divalent metal cations to create octahedrons having linked edges^26–28^. Trivalent cations replace some of the cationic sites, creating a positive charge excess that is balanced by hydrated anions. These compounds are represented by the general formula: [M^2+^_1-x_M^3+^_x_(OH)_2_] ^x+^A^m-^_x/m_.nH2O, where M^2+^ and M^3+^ are the cations involved, and A is an anion of charge m-^29^^,^^30^. With exchangeable interlayer anions retained, the composition’s ultimate result is a hydroxylated structure with positively charged layers^28,29^. Due to their huge specific surface area, low poisoning risk, strong anion substitution capacity, ease of recovery, inexpensive synthesis, and great stabilities for thermal and chemical properties, LDH have garnered a lot of interest as adsorbents in recent years^31^. The type of hybridizing material (adsorbent) and pollutant (adsorbate) mostly determines the adsorption mechanism of the various harmful pollutants on the hybrids. Hydroxide precipitation, Physical adsorption, anion-metal complexes, pi-pi interactions, electrostatic contact, and chemical bonding are some of the most common ways that LDH-containing hybrids stick to things^26^^,^^27^^,^^32^^,^^33^.

Aim of the work: Valorization of adsorbent spent (arsenic, lead, and mercury from industrial wastewater adsorbed by double-layered hydroxide) and investigating the safety and toxicity in vivo study for confirmation the efficacy of LDH in heavy metals removals.

While extensive literature exists on the high adsorption capacity of Layered Double Hydroxides (LDHs) for As^3+^, Pb^2+^, and Hg^2+^ (e.g.,^34^ there is a critical research gap regarding the toxicological profile of the resulting ‘spent’ adsorbents. Most studies focus on removal efficiency, yet the biological risk posed by these metal-laden matrices during handling, transport, or potential soil-amendment valorization remains poorly understood.

We hypothesize that the structural stability of the Zn-Co-Fe/LDH framework significantly mitigates the inherent toxicity of sequestered heavy metals by reducing their bioavailability. To test this, the current study utilizes Probit Analysis, a specialized regression model used in toxicology to transform sigmoid dose–response data into a linear relationship. This allows for the precise calculation of the median lethal dose (LD_50_) and establishes a quantitative safety threshold for industrial waste management. So the prospective outcomes of this research include the establishment of safe-handling dosages for metal-laden LDHs and a comparative toxicity hierarchy (As vs. Pb vs. Hg). These findings will provide a regulatory framework for the sustainable ‘cradle-to-grave’ management of advanced adsorbent materials.

## Materials and methods

### Chemicals

The zinc nitrate [Zn(NO_3_)_2_6H_2_O–extra pure 98%], cobalt nitrate [Co(NO_3_)_2_6H_2_O–extra pure 98%], and iron nitrate [Fe(NO_3_)_3_9H_2_O–extra pure 98%] were purchased from LobaChemie, India. Provided the hydroxide hydrochloric acid and the sodium had been provided from Chem-lab NV. All prepared solutions were made using Milli-Q water with a resistivity of 18.2 MΩ cm.

### Synthesis of Zn-Co-Fe/LDH

**Fig. 1:**
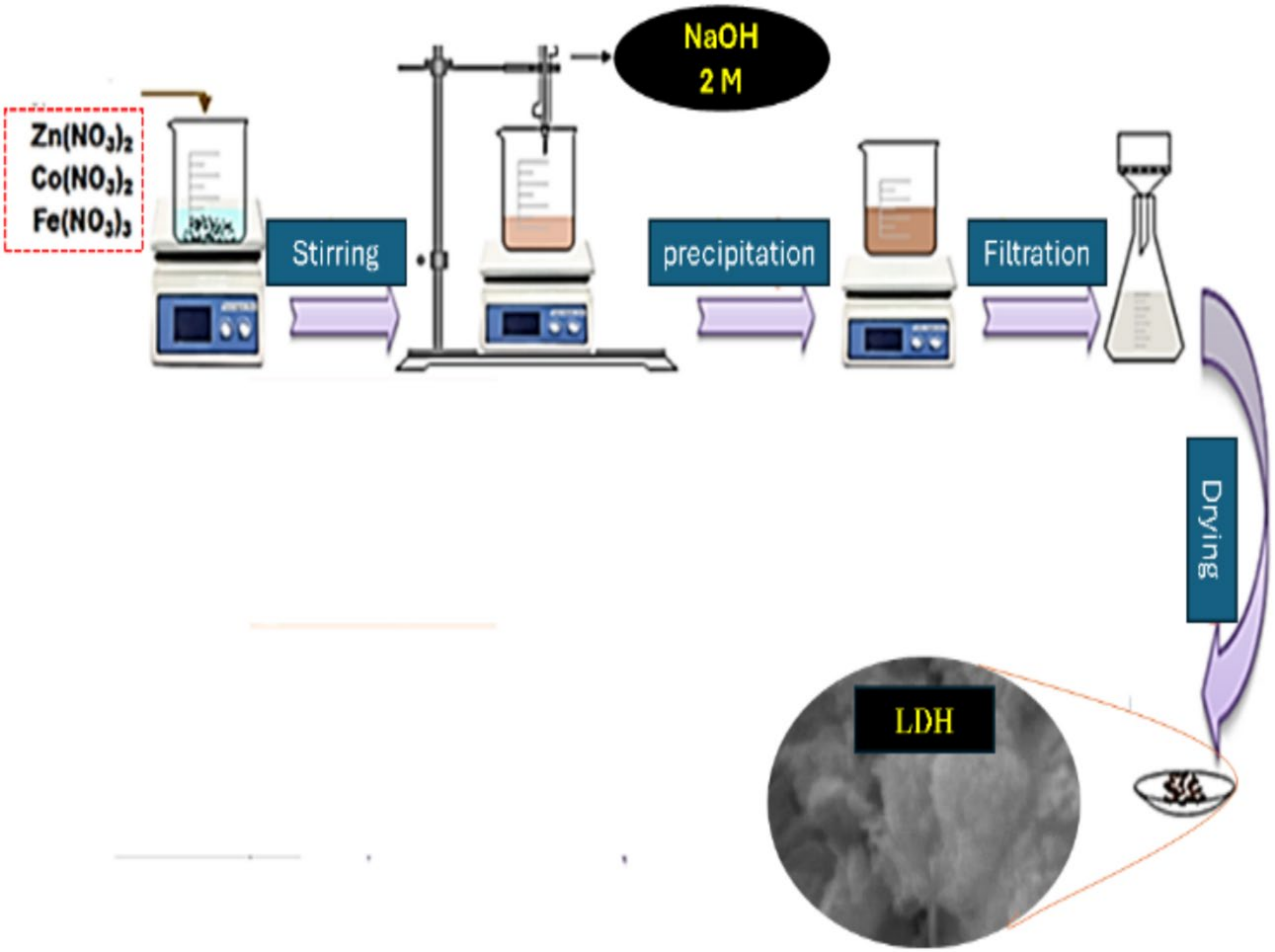
Synthesis procedure of Zn-Co-Fe/LDH.

Co-precipitation was applied to synthesize the Zn-Co-Fe/LDH. Cobalt, Zinc and Iron nitrate salts had been mixed by 2:2:1 molar ratio, and 100 mL of deionized water was dissolved in it while being stirred continuously at room temperature (25 °C). Precipitated using the slow addition (0.1 mL/min) of 2 M NaOH solution until the pH of the solution was reached 10 to guaranty complete precipitation Fig. 1. The resulting sample was aged overnight (24 h) under continuous stirring at room temperature (25 °C). The formed suspension was then filtered and washed several times using distilled water to get rid of excess hydroxide and then washed using ethanol. After that the solid was dried using vacuum dryer for 18 h at 60 °C^35^.

### Characterization of Zn-Co-Fe/LDH

The X-ray diffraction (XRD) patterns of Zn-Co-Fe/LDH before and after adsorption were collected by PANalytical (Empyrean) (Cu Kα source, λ = 0.154 nm). FT-IR spectra were registered using Bruker Vertex 70 (serial number 1341). The morphology of Zn-Co-Fe/LDH was determined using a scanning electron microscope (SEM, JSM-IT200).

### Experiments on adsorption

A stock solution of mortals was created in order to get the required concentration. Several solutions were evolved. For As^3+^, Pb^2+^, and Hg^2+^, the pH levels were set at 3 and 4.5, respectively. In 20 ml of the heavy metal ion solution, LDH was added and dissolved at concentrations of 20 parts per million for As^3+^ and 50 parts per million for Hg^2+^ and Pb^2+^. The flasks were shaken in order to reach equilibrium. The samples were extracted and filtered at predetermined intervals using a 0.45-m PES filter. The number of metal residual ions in the sample was estimated using an inductively coupled plasma (ICP) apparatus^35^.

The removal efficiency (%) and adsorption capacity qe (mg/g) were evaluated using the following equations:

1 $$RE(\%) = \frac{ {C}_{o}-{C}_{e}}{{C}_{o}}\times 100$$

2 $${q}_{e}=\frac{\left({C}_{o}-{C}_{e}\right)*V}{m}$$

where C_o_ and C_e_ are the initial heavy metals concentration and concentration at the equilibrium, respectively (mg/L), V is the volume of the solution (L) and m is the quantity of LDH (g).

### *In-vivo* acute and sub toxicity study

#### Experimental animals

The standard laboratory environment was used to maintain the temperature at 23 °C, the humidity at 60%, and the light/dark cycle at 12 h for the rats that were obtained for this study. The measurement and gavage procedures were evaluated and authorized by the Animal Rights Ethical Treatment Institute by Institutional of Animal Care and Use Committee (IACUC) of Beni-Suef University (BSU) as (BSU-IACUC), with approval number 022–459 dated 22/10/2023. This study was designed, performed, and reported in strict accordance with the ARRIVE guidelines 2.0 (Animal Research: Reporting of in vivo Experiments) for the care and use of laboratory animals. Sample Size Justification, Randomization, Blinding and Outcome Measures. All efforts were made to minimize animal suffering and reduce the number of animals used, in compliance with the principles of the 3Rs.

For the duration of the investigation, a standard 12-h light/dark cycle was implemented. The LD_50_ values were determined by employing a total of one hundred and fifty adult male rats, weighing 180 to 200 g body weight, in toxicological experiments. All animals were provided with a standard laboratory pellet diet and water ad libitum throughout the duration of the acute investigation.

The experimental animals employed in the present investigation were housed at the Faculty of Postgraduate Studies for Advanced Sciences (PSAS). They were maintained in accordance with the Guidelines of Animal Care and the Experimental Animal Ethics Regional Committee at the University of Beni-Suef, Egypt, which granted ethical approval for the experimental work with approval number 022–459.

### Experimental groups and medications for 10 days’ acute study

In this study, one hundred and twenty (120) mature Albino male rats were employed with 180–200 gm weight and 4–6 months of age. For each trial, rats were split into four equal groups of 5 rats which subdivided into six groups for each dosage upgrade. Zn-Co-Fe/LDH before adsorption, after LDH/As, Pb and Hg adsorption which all was begun at 50 mg per kilogram, in the first group and was increased in various groups until it reached 1000 mg per kilogram b.wt.in the last group Table 1.

**Table 1:** Estimations of dead number of animals at different groups.

No. of dead animals	No. of animals/group	Dose (mg/kg b.wt.)	Group
0	5	50	Zn-Co-Fe/LDH
0	5	100	
0	5	200	
1	5	400	
1	5	600	
3	5	800	
5	5	1000	
0	5	50	Zn-Co-Fe/LDH- As
0	5	100	
1	5	200	
2	5	400	
4	5	600	
4	5	800	
5	5	1000	
1	5	50	Zn-Co-Fe/LDH- Pb
2	5	100	
4	5	200	
5	5	400	
5	5	600	
5	5	800	
5	5	1000	
1	5	50	Zn-Co-Fe/LDH- Hg
1	5	100	
2	5	200	
3	5	400	
4	5	600	
5	5	800	
5	5	1000	

### Acute oral toxicity study

A study on the acute oral toxicity has been carried out in compliance with OECD Test Guideline 420^36^. The acute oral toxicity was evaluated following the dosage selection principles of OECD Test Guideline 420, utilizing a series of fixed doses (50, 100, and 200 mg/kg) to observe evident toxicity and mortality. To provide a precise estimation of the median lethal dose, the resulting mortality data were subjected to Probit Analysis. This statistical method transforms the sigmoid dose–response curve into a linear relationship by converting response percentages into Probit units (Y) and doses into logarithmic values (X). The LD_50_ was then derived from the regression equation Y = a + bX, allowing for a quantitative comparison of the toxicity levels between the different metal-laden adsorbents. The sub-acute oral toxicity study was conducted in accordance with the WHO^37^ and (OECD) for chemical investigations^36^.The rats were famished for 16 h prior to the oral administration day. Rats (n = 10) were administered a single oral dose of the nanomaterials as a suspension before and after adsorption, while the control group received only the vehicle. Observations were noted at first 4 h and, for a period of 10 days, once daily to monitor for any indications of acute or subacute toxicity. On a daily basis for a period of 10 days, visual observations were made for any deaths, clinical signs, behavioral changes (e.g., lethargy, salivation), injury, and illness. Behavioral assessments included the monitoring of autonomic profiles (e.g., salivation, lacrimation, and piloerection), neuromuscular changes (e.g., gait abnormalities, tremors, and convulsions), and central nervous system activity (e.g., drowsiness, lethargy, and stereotypy). A qualitative scoring system was employed where: (0) indicated absence, (1) indicated mild presence, and (2) indicated severe manifestation of the clinical sign. These observations were recorded daily to ensure a comprehensive evaluation of the neurotoxic potential of the spent adsorbents.

The acute toxicity study was conducted following a Classical Dose–Response Protocol to facilitate Probit Analysis. While initial dose ranges were informed by the OECD 420 sighting study principles, the main study was expanded to include multiple escalating dose groups. This design was chosen to provide a high-resolution LD50 value, which is a prerequisite for establishing safety benchmarks in the industrial valorization of spent adsorbents.

Ethical Consideration (The 3Rs Justification): All animal procedures were conducted in compliance with the ARRIVE guidelines. To adhere to the principle of Reduction, the animals used for the acute phase also provided the baseline data for the sub-acute biochemical markers, thereby maximizing the data yield per animal and avoiding the need for separate pilot studies.

On the eleventh day, Ketamine: xylazine combinations in a 1:1 ratio, with 90 mg/kg and 5 mg/kg b.wt., respectively was injected intraperitoneally to put all of the animals to sleep. Blood samples were obtained through cardiac puncture and partitioned into EDTA and plain tubes for hematological and biochemical analyses, respectively. Following excision, the liver, heart, stomach, kidneys, and lungs were immediately preserved in 10% buffered formalin for subsequent histopathological examination^38^.

### LD_50_ and LD_90_ are evaluated as a toxicity metric through probit analysis

Experimental data can be collected by the independent variable X Probit values are obtained by converting the value evaluation of a mathematical model that is most appropriate for the dependent variable Y (percent)^39,40^. The LD_50_ was calculated by analyzing the relationship between the dosage (X) and the observed mortality rate (Y) using Probit and regression analyses. This approach offers a more precise toxicological evaluation than traditional percentage-based mortality calculations^40^^,^^30^.

The Miller and Tainter method^41^ was employed to determine the LD _50_ by converting mortality percentages into Probit values. This method is particularly significant when experimental data (Y) includes 0% or 100% mortality at the lowest or highest doses, respectively^42^. In such instances, mortality percentages are corrected based on the number of animals in each group before being transformed into Probit units. This correction allows for the calculation of the LD_50_ and LD_100_ in mg/kg rather than as simple percentages, providing a more precise estimation of acute toxicity.

### LD_100_ and LD_50_ estimation

The test materials were administered orally to the rats at doses ranging from 50 to 200 mg/kg body weight (b.wt.). Following administration, the animals were monitored continuously for the first two hours, and subsequently at 24-h intervals for up to 10 days. The mortality rate (%) was recorded between the 24th hour and the 10th day^43^. Data were analyzed using Probit analysis in SPSS software to determine the linear correlation coefficient and evaluate mortality trends in relation to the administered doses. The linear correlation coefficient was determined using the probit analysis feature of the SPSS program, which also analyzed mortality trends in relation to the measured drug concentrations^44^^,^^43^.

### Symptoms

The animals were monitored daily for a period of 10 days to record mortality and any clinical signs of toxicity, including changes in physical appearance and behavior (e.g., drowsiness, salivation, and lethargy). These observations were performed more frequently during the first 4 h following dosage^45^.

### Hematological investigations

All haematological parameters were estimated as full complete blood picture^46^. Following the collection of blood in non-heparinized containers, sera were collected, creatinine, urea, and liver function enzymes were estimated^47^.

### Biochemical estimations for safety investigation

#### Hematological estimations

Manually, blood samples were mixed with EDTA to conduct relative differential counts and total counts of white blood cells (WBCs) as previously described^48^. Following the initial counts, 80 mg of nylon fiber were incubated with one milliliter of blood sample at 37 °C for 15 min. Total and differential leukocyte counts were performed to determine the proportions of neutrophils, lymphocytes, monocytes, eosinophils, and basophils, following the methodology described by Ghule et al.,^49^.

#### Histopathological investigation

The experiment ended with the rats male rats sacrificed and their livers, hearts, stomachs, lungs, and kidneys dissected, cleaned in normal saline, and immersed in formalin 10% solution. Following 24 h of fixation in 10% buffered formalin, the tissue samples were dehydrated and embedded in paraffin wax. Using increasing graded amounts of ethanol, dehydration, xylene clarifying, soft paraffin impregnation, hard paraffin embedding, and blocking were performed. The paraffin-embedded tissue blocks were sectioned at a thickness of 2–5 µm using a rotary microtome.

Prior to H&E staining, these segments were put on clean. Stained sections were examined using a light microscope with 10 X and 20 X objective lenses and an LEICA (DFC290 HD system digital camera, Heerbrugg and Switzerland)^50^. Beni-Suef University faculty of veterinary medicine histopathology laboratory performed the histological investigation. They were fixed in 10% neutral buffer formalin, trimmed, washed, dehydrated in various concentrations of ethyl alcohol, purified in xylene, and embedded in paraffin. A hematoxylin and eosin stain was applied to thin slices (4-6µm)^50^.

#### Statistical investigations

The regression analysis was conducted using Excel (IBM SPSS Statistic 26.0, Armonk, NY, USA), while the statistical analysis was conducted using SPSS (version 20.0) software. A P value of less than 0.05 was considered statistically significant^51^.

Data were first subjected to the Shapiro–Wilk test to assess normality and the Levene’s test to confirm homogeneity of variance. For normally distributed data, differences between the control and multiple treatment groups were analyzed using One-Way Analysis of Variance (ANOVA), followed by Tukey’s Honestly Significant Difference (HSD) post-hoc test for pairwise comparisons. Data are expressed as mean ± standard deviation (SD). A p-value of **p < 0.05** was considered statistically significant, and exact p-values are reported for all critical biochemical and hematological parameters. Sample sizes and error bar definitions are provided in the respective figure legends.

Randomization and Blinding: To minimize selection bias, 150 Sprague–Dawley rats were assigned to five experimental groups using a simple randomization technique based on a computer-generated random number sequence. Each group was balanced for initial body weight (mean ± SD) to ensure homogeneity. A single-blind strategy was implemented during the histopathological and biochemical assessments; the veterinary pathologists and laboratory analysts were blinded to the treatment identifiers (e.g., LDH-As vs. LDH-Pb) until the completion of the data analysis phase to ensure objective interpretation of the results.

The sample size was determined using a power analysis (G*Power version 3.1.9.7). Based on preliminary data for heavy metal-induced mortality, a total sample size of 150 rats (n = 6 per dose level across five escalating doses per treatment group) was calculated to achieve a statistical power (1-beta) of 0.80 and a significance level (alpha) of 0.05. This cohort size was specifically chosen to provide sufficient sensitivity for Probit regression analysis and to allow for the simultaneous evaluation of sub-acute biochemical markers without requiring additional animals, thereby adhering to the principle of Reduction within the 3Rs framework.

## Results and discussions

### Characterizations of adsorbent

#### Fourier transform-infrared spectrometry of Zn-Co-Fe/LDH before and after metal ions adsorption

**Fig. 2:**
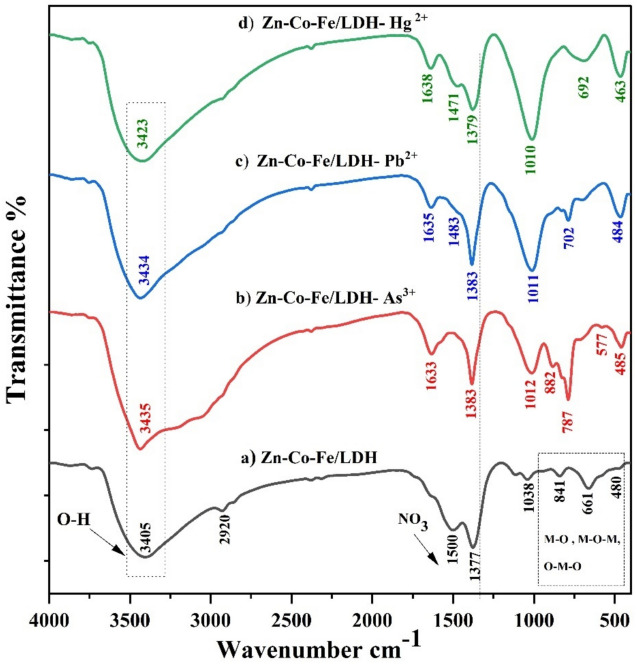
FT-IR spectra of the **a**) Zn-Co-Fe/LDH, **b**) Zn-Co-Fe/LDH after adsorption of As^3+^, **c**) Zn-Co-Fe/LDH after adsorption of Pb^2+^, and **d**) Zn-Co-Fe/LDH after adsorption of Hg^2+^.

The FT-IR spectrum of Zn-Co-Fe/LDH is depicted in Fig. 2a. The absorption bands observed below 1000 cm^-1^ include 814 cm^-1^, 661 cm^-1^, and 480 cm^-1^ correspond to the stretching and bending vibrations of the LDH structure (M–O, O–M–O, and M–O–M), where M denotes the metal ions Zn, Co, or Fe^52–54^. In the spectrum of Zn-Co-Fe/LDH, a prominent band appears around 1377 cm⁻^1^, attributed to the asymmetric stretching of NO₃⁻ within the LDH structure^55^. Additionally, the band at 3405 cm⁻^1^ is assigned to the stretching vibrations of hydroxide ions in adsorbed water and interlayer water molecules^56,57^. Figure 2(a-d) shows the LDH’s FTIR spectra after the adsorption of As^3+^, Pb^2+^, and Hg^2+^ heavy metals. The spectra exhibited their characteristic bands *albeit* with significantly reduced intensity, demonstrating the maintains of the LDH’s chemical structure and the occurrence of an anion-exchange reaction between Zn-Co-Fe/LDH and As^3+^, Pb^2+^, and Hg^2+^ heavy metals^58^. Notable disparities were observed in the positioning of the absorbance peaks. Specifically, the band associated with hydroxyl groups displayed an approximate shift from 3405 to 3435, 3434, and 3424 cm^-1^ for As^3+^, Pb^2+^, and Hg^2+^, respectively. Additionally, the peak associated with nitrate groups (NO₃⁻) at 1377 cm^-1^ demonstrated slight shifts to 1383, 1383, and 1379 cm^-1^ for As^3+^, Pb^2+^, and Hg^2+^, respectively, signifying modifications in the M-NO_3_ bond during the adsorption process^59^. Moreover, the peaks below 1000 cm^-1^ experienced significant shifts with slightly increases in intensity upon heavy metal adsorption, which can be attributed to the displacement of metal ions through surface complexation^60^^,^^61^.

#### Mechanism of heavy metal adsorption

The desorption method (adsorbate ions are transported from the adsorbent surface through reverse adsorption) can be used to mimic adsorption for a variety of applications because it is a reversible process under certain conditions. Adsorption onto a solid adsorbent is accomplished by three primary processes: the pollutant is carried from an aqueous solution to the adsorbent’s surface, adsorption takes place on the solid surface, and the pollutant is carried inside the adsorbent particle. The mechanism of heavy metal adsorption onto LDH is can be summed up as follows: surface complexation, surface precipitation, ion exchange, including As^3+^, Pb^2+^, and Hg^2+^, and non-electrostatic attraction. FTIR analysis may be used to better explore the adsorption mechanisms of heavy metals. FTIR analysis may be used to better explore the adsorption mechanisms of lead. The heavy metals and the interlayer anions exchanged ions. The peak at 1377 cm^-1^ was slightly shifted to 1383, 1393, and 1379 cm^-1^ following the adsorption of As^3+^, Pb^+2^, and Hg^2+^, indicating a modification in the M-NO_3_ bond. The hydroxyl groups at the active sites were swapped out for heavy metal, which was then discharged into the solution. The band’s strength at 1044 and 846 cm^-1^ was significantly altered and diminished during adsorption. This is because surface complexation causes As^3+^, Pb^+2^, and Hg^2+^ to be displaced and the original M–O bond to break, reducing the M–O’s stretching vibration intensity. One of the main techniques for removing inorganic contaminants from adsorbents is the formation of mineral precipitates in the solution or on the sorbing material’s surface. A particular kind of interaction known as "monodentate attraction of heavy metals with LDHs" occurs when a heavy metal ion bonds with a single donor atom on the LDH’s surface. A heavy metal ion (As^3+^, Pb^2+^, and Hg^2+^) can connect with an oxygen atom of a hydroxyl group in the LDH structure by this contact, which is a type of non-electrostatic attraction. The LDH adsorbed sample’s FTIR spectrum demonstrates that following As^3+^, Pb^+2^, and Hg^2+^ adsorption, respectively, the intensity of -OH (3405 cm^-1^) shifts significantly to wavenumbers 3445, 3434, and 3423 cm^-1^.

#### X-ray diffraction analysis of Zn-Co-Fe/LDH after metal ions adsorption

**Fig. 3:**
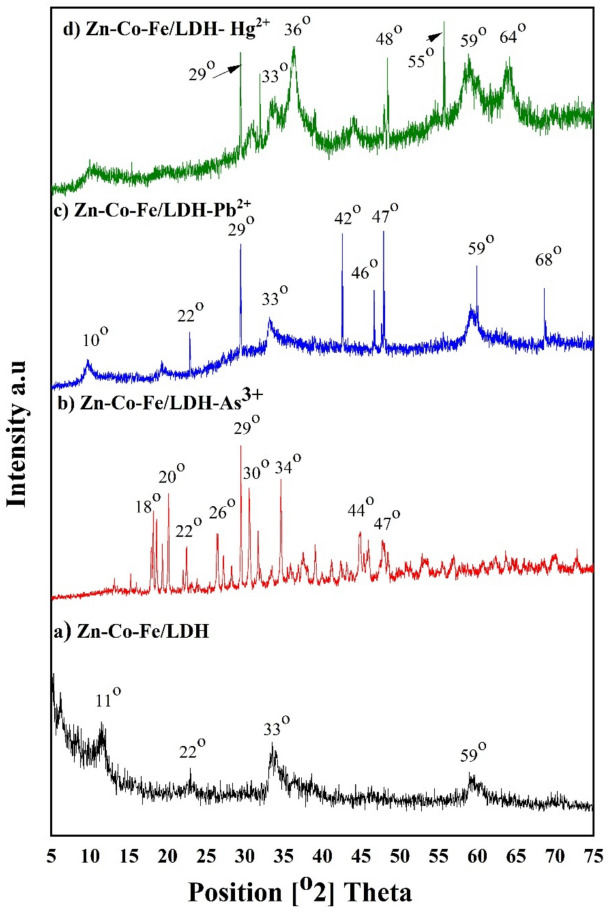
XRD spectra of the **a**) Zn-Co-Fe/LDH, **b**) Zn-Co-Fe/LDH after adsorption of As^3+^, **c**) Zn-Co-Fe/LDH after adsorption of Pb^2+^, and **d**) Zn-Co-Fe/LDH after adsorption of Hg^2+^

The X-ray diffraction (XRD) patterns of Zn-Co-Fe/LDH, shown in Fig. 3a, display the characteristic reflections of layered double hydroxide (LDH) materials. The prominent peaks observed at 2θ values of 11.5°, 22.7°, 33.6°, and 59.2° correspond to the (003), (009), (011), and (110) crystal planes, respectively. These reflections are consistent with standard reference codes (04–018-3495) and (JC-PDF No. 32–1476), confirming the structural integrity and phase purity of the synthesized LDH. These findings, supported by previous studies^53,54,62,63^, confirmed the structural composition of Zn-Co-Fe/LDH. The XRD analysis indicated that the pattern exhibited well-defined crystalline LDH properties. Using the Debye–Scherrer formula, the crystallite size of the Zn-Fe-Co/LDH was calculated to be 99.25 Å. Additionally, the material exhibited a basal spacing of 2.66 Å, which is indicative of the interlayer distance within the LDH structure.

Figure (3b-d) shows the Zn-Co-Fe/LDH XRD patterns after the adsorption of As^3+^, Pb^2+^, and Hg^2+^ heavy metals. Upon comparing the Zn-Co-Fe/LDH XRD pattern before and after the adsorption of these heavy metals, several observations can be made. Overall, the XRD patterns of Zn-Co-Fe/LDH after the adsorption displayed enhanced crystallinity, accompanied by the emergence of additional peaks. Besides, the intensity of some peaks in the Zn-Co-Fe/LDH patterns decreased, while some peaks experienced a shift. While the other peaks remained unchanged throughout the adsorption process. This is likely due to the adsorption process, which could lead to the ordering of the LDH layers as the metal ions interact with the surface. The emergence of new peaks at specific 2θ values suggests the formation of new crystalline phases or a change in the crystal structure upon adsorption. The X-ray diffraction (XRD) patterns of Zn-Co-Fe/LDH after the adsorption of As^3+^ are illustrated in Fig. 2b. Several notable changes can be observed. Additional peaks emerged at 2θ values of 18°, 20°, 26°, 29°, 30°, 44°, and 47°, while the peak at 33° shifted to 34°. Furthermore, the peaks at 11° and 59° disappeared. Similarly, the XRD patterns of Zn-Co-Fe/LDH after the adsorption of Pb^2+^, as showed in Fig. 2c, exhibited distinct alterations. Additional peaks emerged at 2θ values of 29°, 42°, 46°, 47°, and 68°, while the peak at 10° shifted to 11°. Figure 2d showed the XRD patterns of Zn-Co-Fe/LDH after adsorption of Hg^2+^. Additional peaks appeared at 2θ values of 29°, 36°, 48°, 55°, 30°, and 64°, while the peak at 11° shifted to 10°. Additionally, the peak at 22° completely disappeared.

On other hand, the crystallite size of the Zn-Fe-Co/LDH after adsorption heavy metals was calculated to be 616.18 Å, 253.32 Å, and 204.06 Å for Zn-Fe-Co/LDH-As^3+^, Zn-Fe-Co/LDH-Pb^2+^, and Zn-Fe-Co/LDH-Hg^2+^, respectively. Moreover, the basal spacing exhibited variations after the adsorption process, transitioning from 2.66 Å to 3.02 Å, 3.03 Å, and 2.80 Å for Zn-Fe-Co/LDH-As^3+^, Zn-Fe-Co/LDH-Pb^2+^, and Zn-Fe-Co/LDH-Hg^2+^, respectively. The increasing of basal space after adsorption demonstrated that heavy metals is interacted with the LDH layers^35^^,^^64^^,^^65^. The heavier metal ions (As^3^⁺, Pb^2^⁺, Hg^2^⁺) are likely accommodated between the layers of the LDH, leading to an expansion of the interlayer distance. The incorporation of these metal ions can result in the modification of the LDH’s layered structure, as seen from the variation in basal spacing. This intercalation could involve both ion exchange and coordination bonding between the metal ions and the hydroxyl or oxygen atoms in the LDH structure.

#### Scanning electron microscopy and EDX of Zn-Co-Fe/LDH before and after metal ions adsorption

**Fig. 4:**
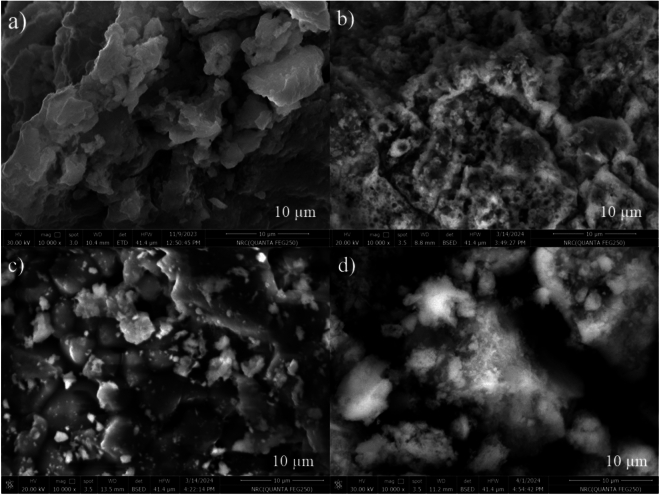
SEM of the **a**) Zn-Co-Fe/LDH, **b**) Zn-Co-Fe/LDH after adsorption of As^3+^, **c**) Zn-Co-Fe/LDH after adsorption of Pb^2+^, and **d**) Zn-Co-Fe/LDH after adsorption of Hg^2+^.

SEM image of the LDH sample that was syntheses is presented in Fig. 4a. The image reveals the surface characteristics of the LDH, which appears to have a uniform surface with multiple layers. It is evident that the LDH surface is homogeneous and exhibits a plate-like structure stacked on top of each other^62^^,^^66^. In Fig. 4a, the EDX elemental mapping of the Zn-Co-Fe/LDH is shown. This mapping demonstrates that the Zn, Co, Fe, N, and O atoms are evenly distributed throughout the entire structure. These observations offer insightful information about the structural and compositional aspects of the sample.

**Fig. 5:**
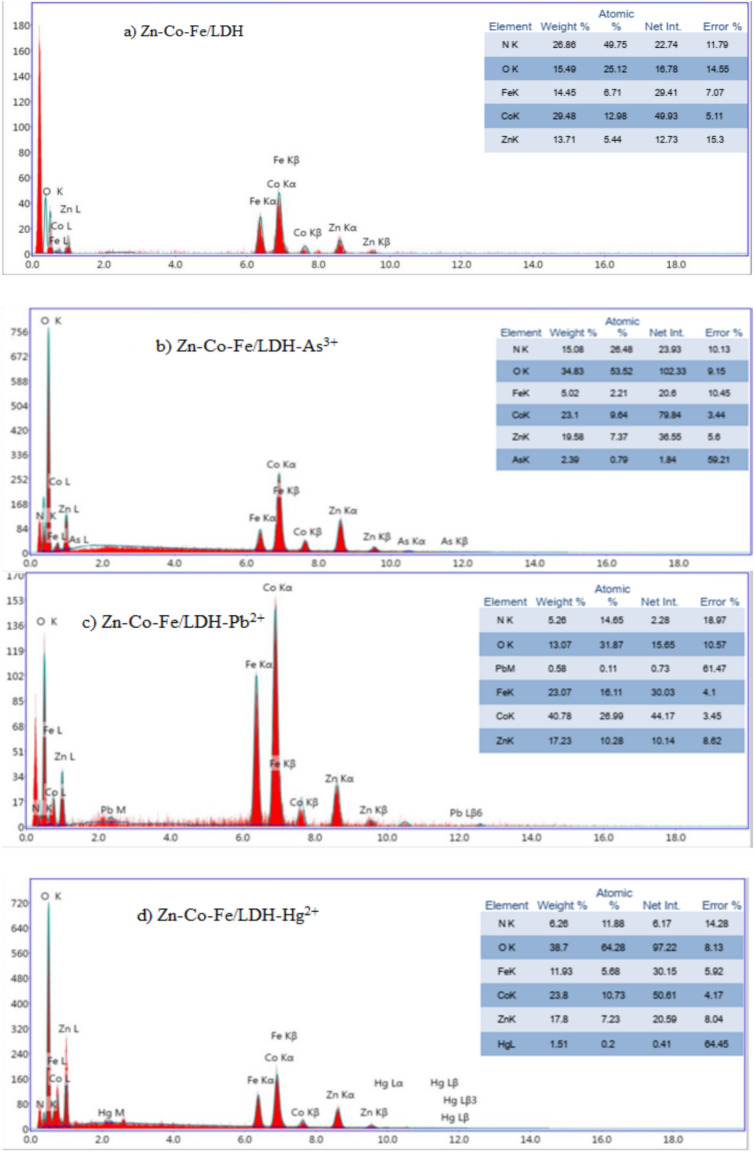
EDX of the **a**) Zn-Co-Fe/LDH, **b**) Zn-Co-Fe/LDH after adsorption of As^3+^, **c**) Zn-Co-Fe/LDH after adsorption of Pb^2+^, and **d**) Zn-Co-Fe/LDH after adsorption of Hg^2+^. Inset figure represents the quantitative percent for elements.

Figure (4b-d) shows the SEM images Zn-Co-Fe/LDH after the adsorption of As^3+^, Pb^2+^, and Hg^2+^ metal ions. A comparison of the surface morphology of LDH both before to and upon adsorption of these heavy metals confirms their successful adsorption. The surface morphology of LDH undergoes a complete transformation after the adsorption, as the interspaces within the LDH layers become partially filled with the heavy metals. The previously homogeneous LDH structure experiences a significant decrease in homogeneity, resulting in a slightly rougher surface appearance. This roughening, observed in the SEM images, is indicative of metal ion interactions with the Zn-Co-Fe/LDH surface. The morphological change is attributed to both the intercalation of metal ions into the LDH layers and surface complexation, where As^3^⁺, Pb^2^⁺, and Hg^2^⁺ replace the original cations and bind to surface active sites. The increased surface roughness enhances the available surface area, potentially facilitating further adsorption and reflecting the structural modifications caused by metal ion incorporation. The EDX elemental mapping of Zn-Co-Fe/LDH subsequent to the sorption of As^3+^, Pb^2+^, and Hg^2+^ heavy metals, as observed in Fig. 5b-d, demonstrates the successful adsorption of As^3+^, Pb^2+^, and Hg^2+^ heavy metals. In Fig. 4b, after the adsorption of As^3+^, Zn-Co-Fe/LDH exhibited the same metal compositions as LDH, along with the presence of As^3+^ at an atomic percentage of 0.79%. Similarly, Figs. 5c and 4d yielded comparable results for Zn-Co-Fe/LDH subsequent to the sorption of Pb^2+^ and Hg^2+^, with atomic percentages of 0.11% and 0.20% respectively. The results obtained offer more proof of the efficient adsorption of heavy metals.

### N₂ adsorption–desorption isotherm analysis

**Fig. 6:**
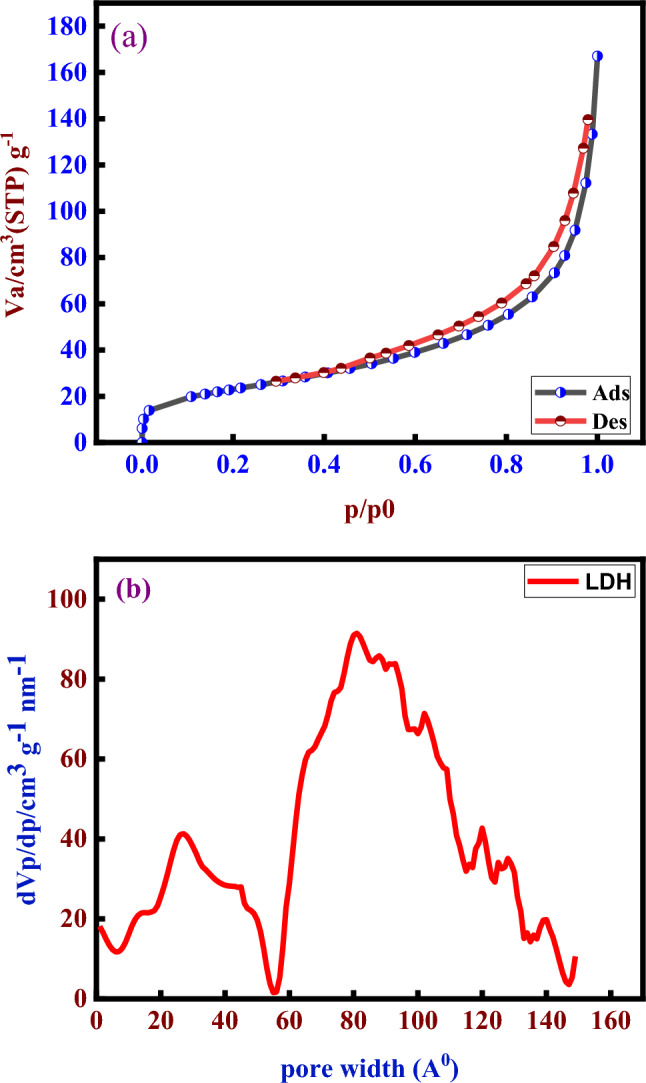
BET adsorption–desorption isotherms (**a**) ZnCoFe/LDH and (**b**) pore width distribution of he prepared material.

The N2 adsorption–desorption isotherms on the sample surfaces are illustrated in Fig. 6 a. Figure 6 b also illustrates the pore size distribution (PSD) curves. Table 2 summarises the surface characteristics obtained from the N2 adsorption–desorption isotherms.

**Table 2:** The surface characteristics obtained from the N2 adsorption–desorption isotherms.

Parameters	LDH
*V*_*m*_[cm^3^(STP) g^-1^]	18.648
*a*_*s,BET*_[m^2^ g^-1^]	81.165
*C*	262.92
Total pore volume (*p*/*p*_0_ = 0.9900) [cm^3^ g^-1^]	0.211
Average pore diameter [nm]	10.401

The acquired isotherms of Zn-Co-Fe/LDH exhibit IV isotherms with pronounced hysteresis loops, characteristic of mesoporous structures.The Zn-Co-Fe/LDH, before to and after to adsorption, displays H3 hysteresis loops, indicative of the clustering of plate-like particles with slit-shaped holes^67^. The inflection points at P/P0 = 0.95 in a sorption–desorption isotherm study indicate the shift from monolayer to multilayer production. Sorption–desorption isotherm analysis often signifies the attainment of the first monolayer of adsorbate on the adsorbent’s surface. At low relative pressures, the adsorbate molecules exhibit a great affinity for the adsorbent’s surface. A monolayer is formed, and the adsorption rate is rapid. As the relative pressure nears 0.9, the surface gets progressively coated with the initial monolayer, signifying a shift in the adsorption dynamics. The surface is approaching saturation, resulting in a shift in the rate of adsorption. This indicates that the majority of the accessible surface area has been filled by the adsorbed molecules, and supplementary gas molecules are starting to populate the multilayer. The adsorption rate diminishes due to a reduced number of accessible adsorption sites^68^^,^^69^. The Zn-Co-Fe/LDH has a larger surface area prior to adsorption.

### Safety and toxicological investigations

#### In-vivo cytotoxicity study

After oral treatment, the acute toxicity of Zn-Co-Fe/LDH at various forms by As^3+^; Pb^2+^; & Hg^2+^ in rats was studied for 10 days. Symptoms of toxicity that were alleviated by mortality included stupor, convulsions, an arched back, rapid respiration, and tremors. The likelihood of mortality began to increase around 300, 100, 30 &20 mg/kg b.wt after oral administration of LDH, LDH- As, Pb & Hg respectively. Number of dead animals at each treatment were recorded at Table 1. The LD_50_ was discovered to be 661.43, 370, 103.7 and 204 mg/kg respectively and (LD_100_) was reached at 1599, 1612, 503, and 2394 mg/kg b.wt. As observed in the Table 3. These findings showed that both Zn-Co-Fe/LDH pre and post adsorption by As^3+^ may be used safely in pharmacological research. For any biological applications or as a therapeutic dose, we used LD_50_ values of 1/20 for the different tested materials. Toxicity increased when the medicine dose was increased in the trial, as seen in Table 4.

- No severe clinical signs (score 2) were observed at the 50 mg/kg dose."
- Mild lethargy (score 1) was noted in the 200 mg/kg group for LDH-Pb during the first 4 h."

**Table 3:** LD_50_ and LD_90_ estimation of Zn-Co-Fe/LDH, before and after adsorption of As, Pb and Hg respectively.

Treatment	LD_50_ (%) (LC_50_)	95% CL		LD_90_ or LC_90_	95% CL		X2 (df = 19)	P*
		LCL	UCL		LCL	UCL		
Zn-Co-Fe/LDH	661.43	463.1	953.7	1075	809.5	504	2.5	0.7
Zn-Co-Fe/LDH- As	370	233	577	859	587	2523	5	0.9
Zn-Co-Fe/LDH- Pb	103.7	50.29	175.34	248	152	1268	5	0.9
Zn-Co-Fe/LDH- Hg	204	95	357	792	433	4009	5	0.7

LCL: lower confidential limit, UCL: upper confidential limit, *X*^*2*^: Chi-square, df: degree of freedom, LC_50_ and LC_90_ were lethal concentration at which 50% and 90% population dies respectively. * *p* > 0.05 is non-significant.

**Table 4:** Safety parameters by Probit analysis.

	Zn-Co-Fe/LDH	Zn-Co-Fe/LDH- As	Zn-Co-Fe/LDH- Pb	Zn-Co-Fe/LDH- Hg
LD_0_	273	97.6	21.36	17.4
LD_20_	303	115.1	25.7	23.3
LD_50_	661.43	370	103.7	204
LD_90_	1075	859	248	248
LD_100_	1599	1612	503	2394

All values are expressed in milligrams per kilogram of body weight (mg/kg b.wt.). LD_50_ and LD_90_ represent the doses required to cause mortality in 50% and 90% of the test population, respectively. LD_0_, LD_20_, & LD_100_ represent the doses required to cause mortality in 0%, 20% and 100% of the test population, respectively.

LD_50_ was calculated and measured based on these investigations and probit analysis. The therapeutic doses for use in biomedical research would be generated from the LD_50_ data as 1/20 of the estimated LD_50_:

$Zn - Co - Fe/LDH\, = = = = \,LD50\, = \,661.43\, \times \,1/20\, = \,33.1$ chosen dose; 33 mg/kg for any biomedical investigations

$${\mathrm{Zn}} - {\mathrm{Co}} - {\mathrm{Fe}}/{\mathrm{LDH}} - {\text{ As}}{-}{-}{\mathrm{LD}}_{{{5}0}} \, = \,{37}0\, \times \,{1}/{2}0\, = \,{18}.{5} {\mathrm{mg}}/{\mathrm{kg}}$$

$$Zn - Co - Fe/LDH - \, Pb{-}{-}LD_{50} \, = \,103.7\, \times \,1/20\, = \,5.2 mg/kg$$

$$Zn - Co - Fe/LDH - \, Hg{-}{-}LD_{50} \, = \,204\, \times \,1/20\, = \,10.2mg/kg$$

**Fig. 7:**
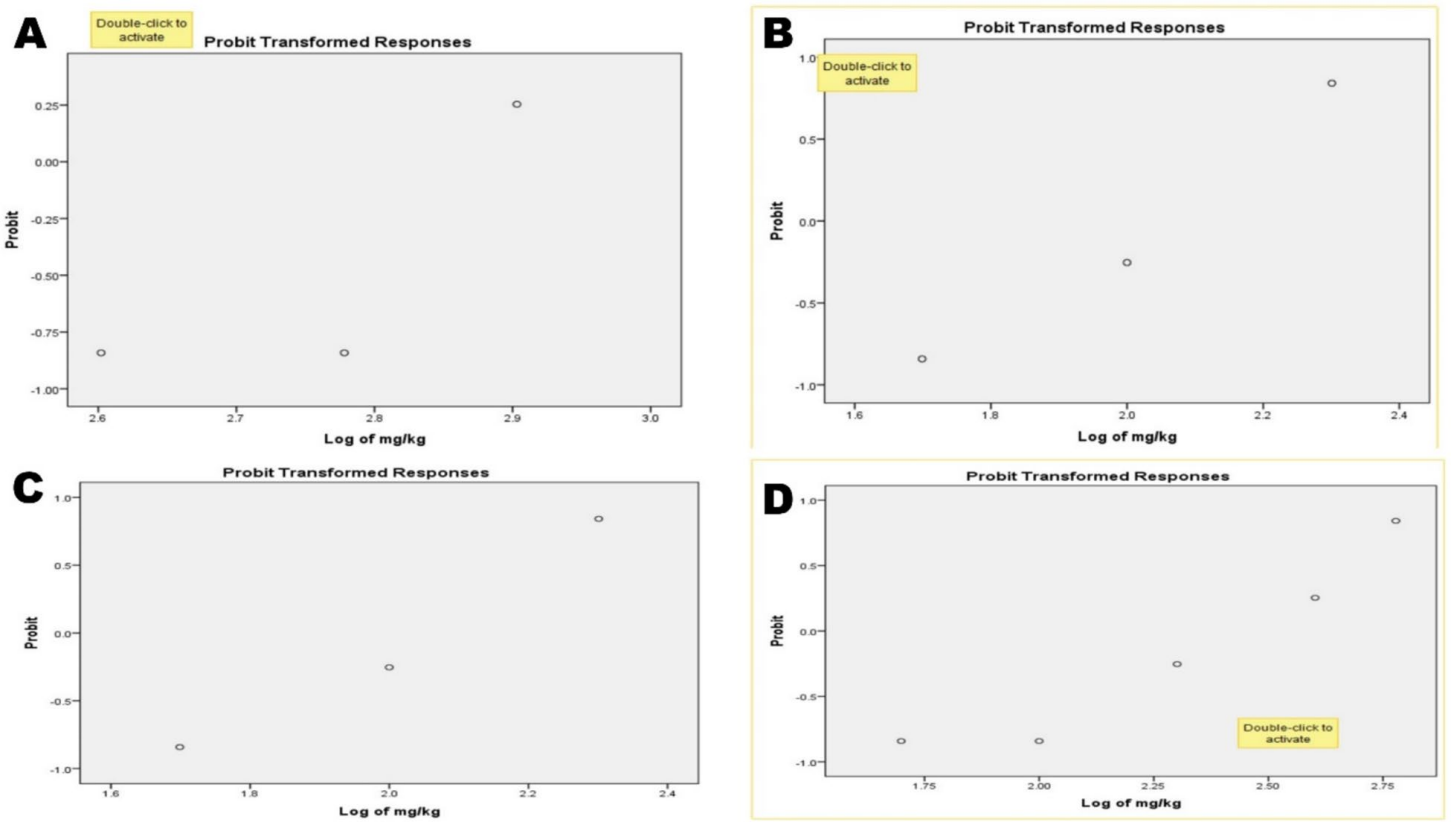
Graphical illustrates for the Log phase of the LD_50_ in different groups, before adsorption by LDH (**A**), after As adsorption (**B**), after Pb adsorption (**C**), & after Hg adsorption (**D**).

**Fig. 8:**
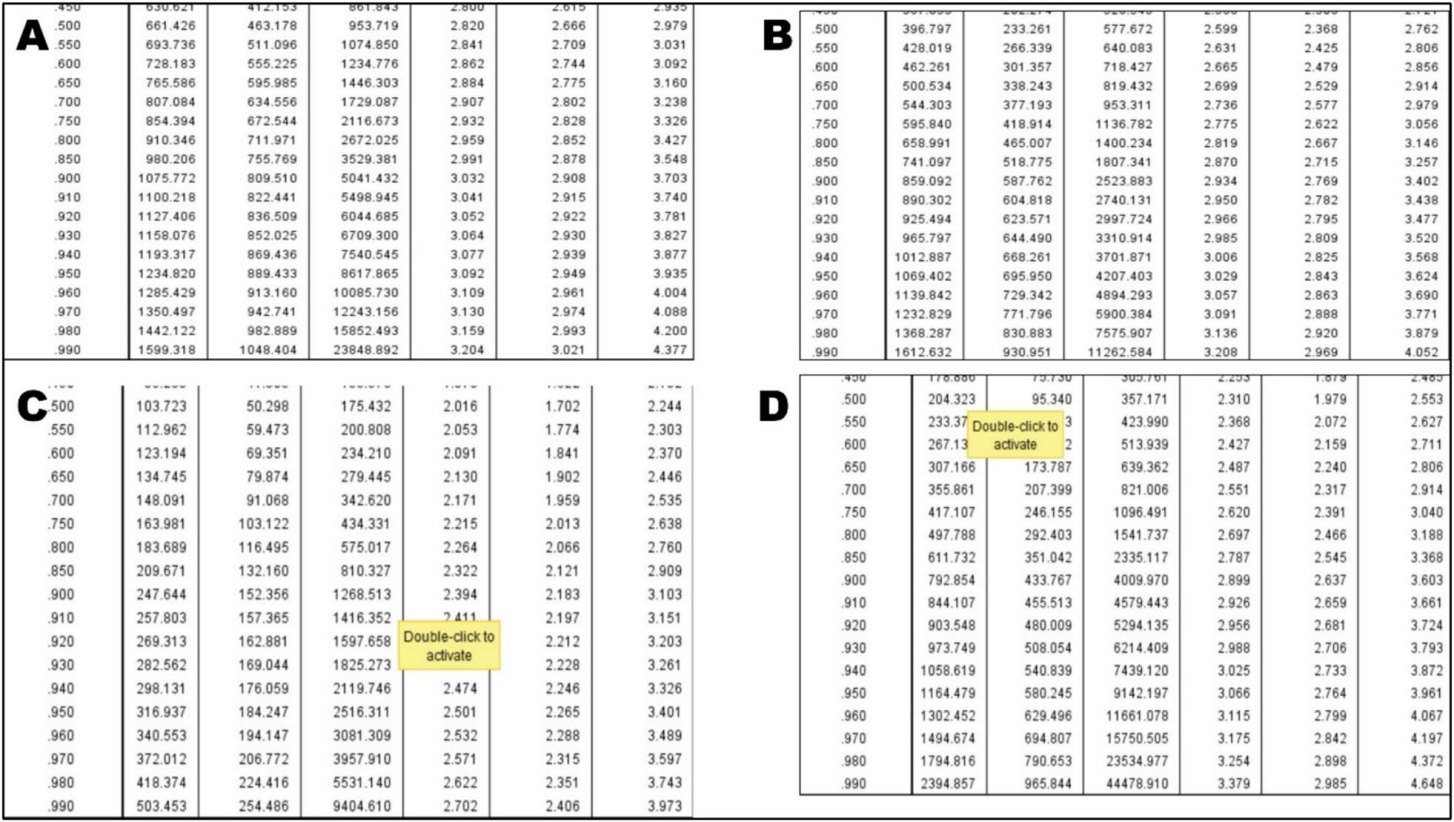
Probit analysis data of the LD_50_ in different groups, before adsorption by LDH (**A**), after As adsorption (**B**), after Pb adsorption (**C**), & after Hg adsorption (**D**).

LD_50_ was estimated and measured from these studies and through the Probit analysis. The safety and potential for further use in biomedical applications of the therapeutic doses that were tested were determined and calculated. Depending on the LD_50_ results. This indicates the highly significant safety of Zn-Co-Fe/LDH, both before and after adsorption for arsenic, as well as after adsorption by Pb and Hg. Therefore, this verified the safety of LDH in various bodily functions. The toxicity was observed following chelation with Pb and hg, and to a lesser extent with, which suggests that LDH is effective in removing heavy metals from water. Also graphical illustrates for the Log phase of the LD50 in different groups, before and after adsorption was illustrated in Fig. 7 while the probit analysis data of the LD50 in different groups, also before and after adsorption was supplied in Fig. 8.

**Fig. 9:**
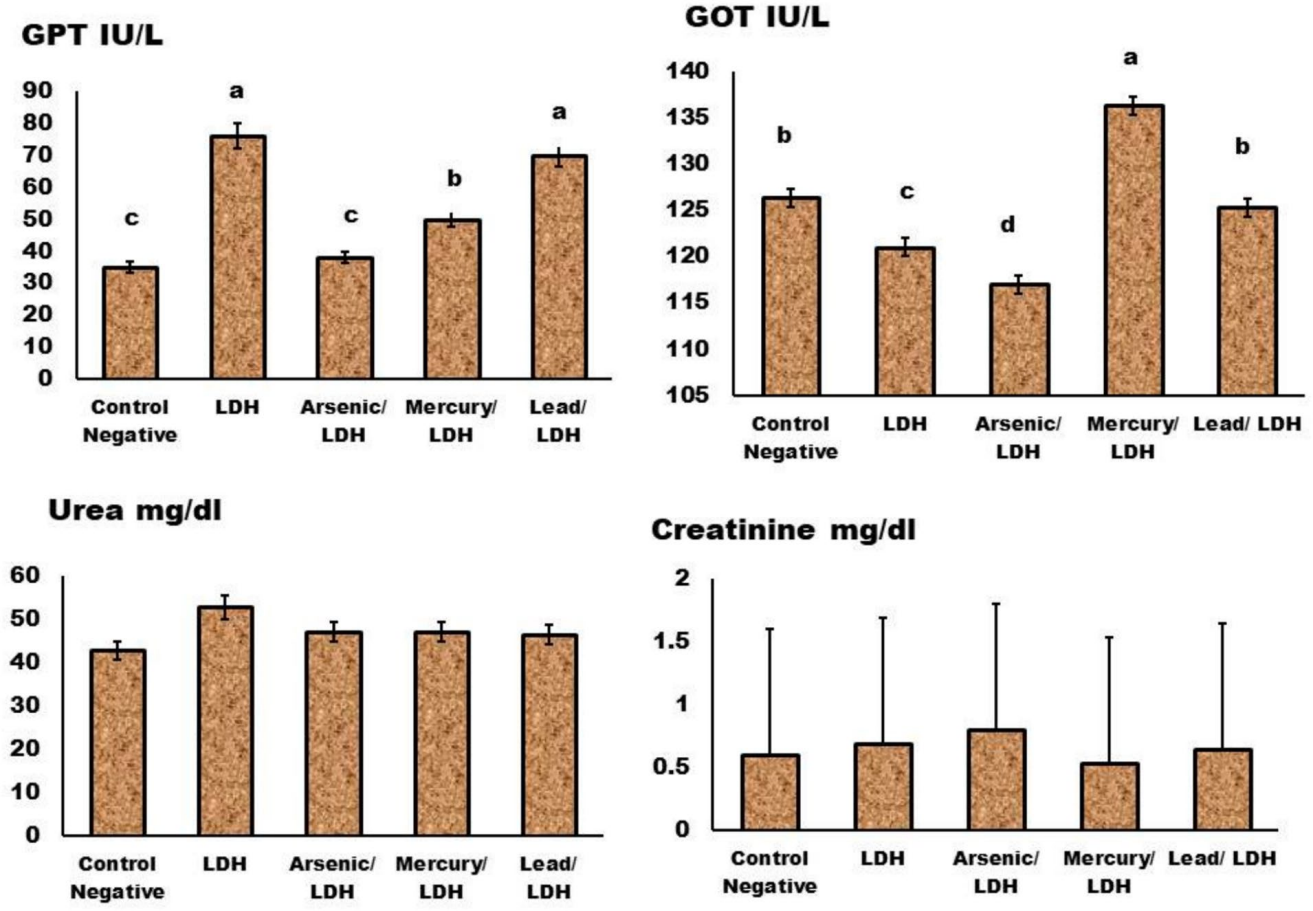
Effect of LDH, alone or after loading with As, Hg and pb on the liver enzymes function (GPT & GOT) & kidney function (Urea and creatinine) levels in serum. Data are presented as mean ± SD (n = 6 per group). Significant differences compared to the control are indicated by letters as determined by One-Way ANOVA followed by Tukey’s post-hoc test." At liver functions ^*a*^*P* < *0.05* when compared to control negative rats; ^*b*^*P* < *0.05* when compared to LDH group, ^*c*^*P* < *0.05*; compared to other types of LDH. No significant differences observed in between different groups with p value > 0.05 at kidney functions.

Liver function was determined as (ALT and AST) (GPT & GOT) blood levels following LDH, which was accompanied by metals adsorption in both enzymes (lead) (Fig. 9). A non-significant increase (*p* > *0.05*) in kidney functions and a significant decrease in blood urea levels indicate a non-nephrotoxic effect that is comparable to that of the control rats (Fig. 9).

**Fig. 10:**
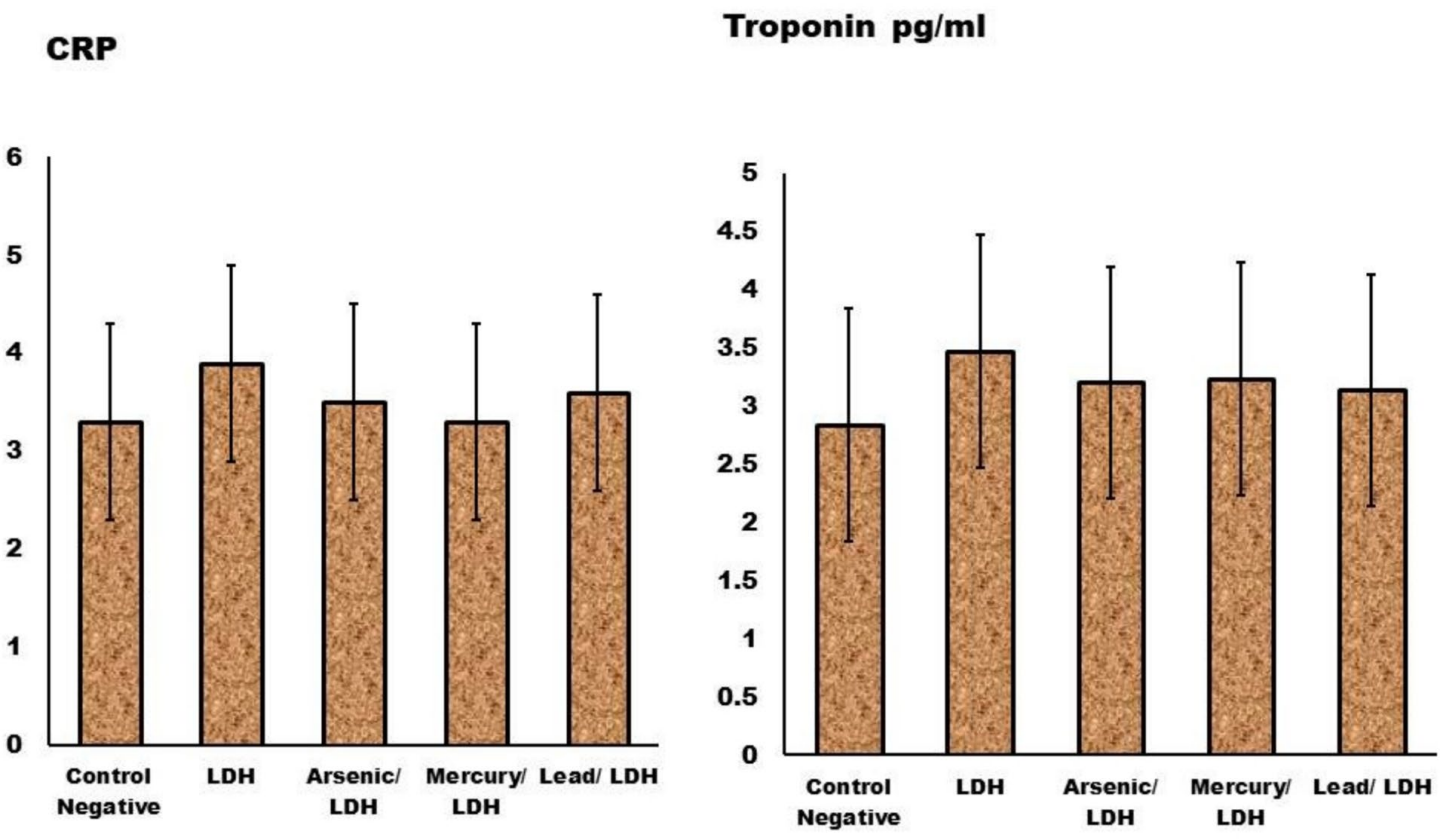
Effect of LDH, alone or after loading with As, Hg and pb on the C—reactive protein (CRP) and Troponin levels in serum Fig 10, no significant differences observed in between different groups with p value > 0.05. Data are presented as mean ± SD (n = 6 per group). Significant differences compared to the control are indicated by letters as determined by One-Way ANOVA followed by Tukey’s post-hoc test.

About inflammatory signs and heart function were represented by (CRP & troponin) levels respectively. Non-significant increase (*p* > *0.05*) in CRP and heart functions indicative its safety without any damage on heart functions as showed in Fig. 10.

**Fig. 11:**
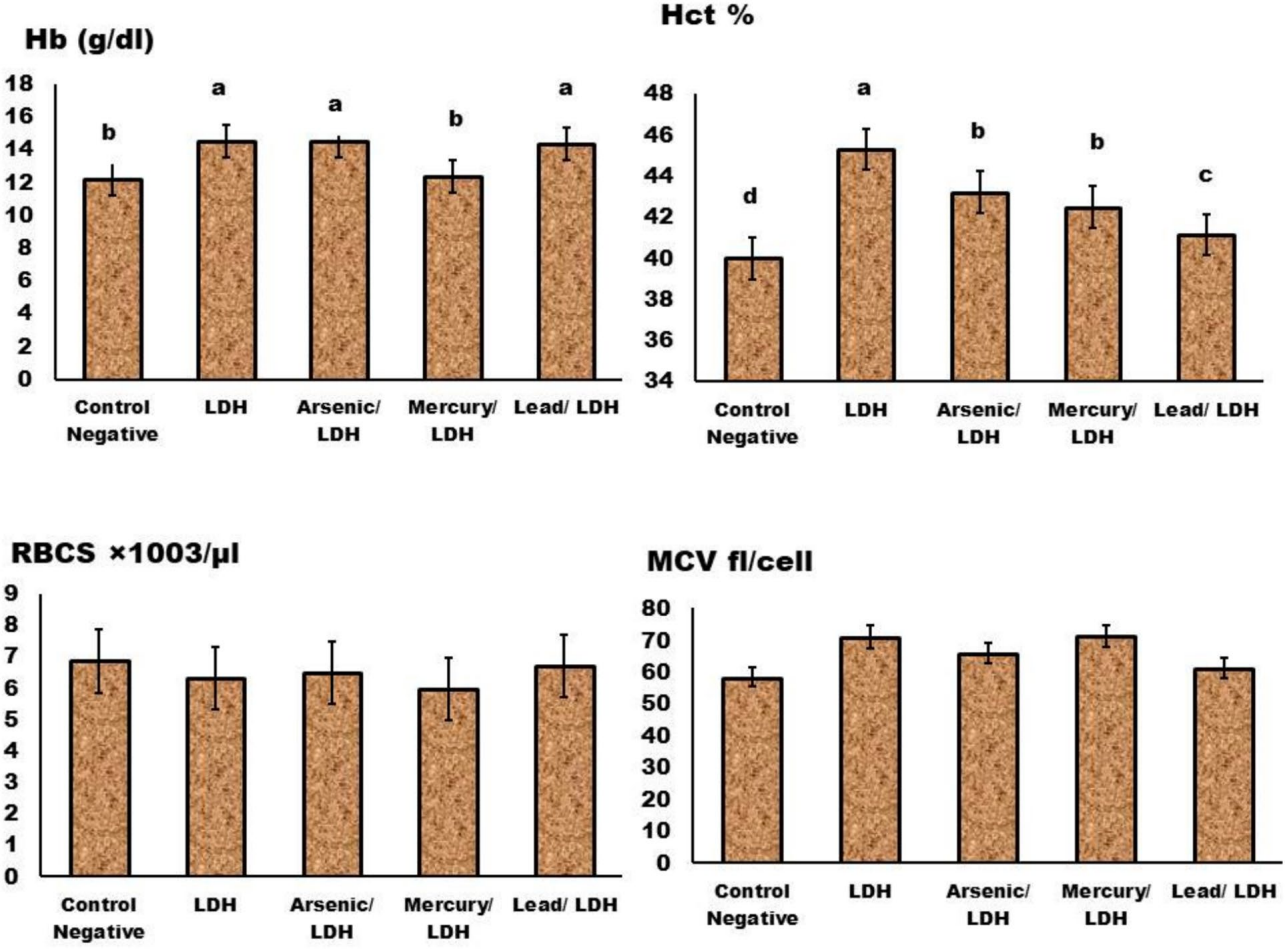
Effect of LDH, alone or after loading with As, Hg and pb on Hb, HCT, RBCs, & MCV, no significant differences observed in between different groups with p value > 0.05. Data are presented as mean ± SD (n = 6 per group). Significant differences compared to the control are indicated by letters as determined by One-Way ANOVA followed by Tukey’s post-hoc test.

**Fig. 12:**
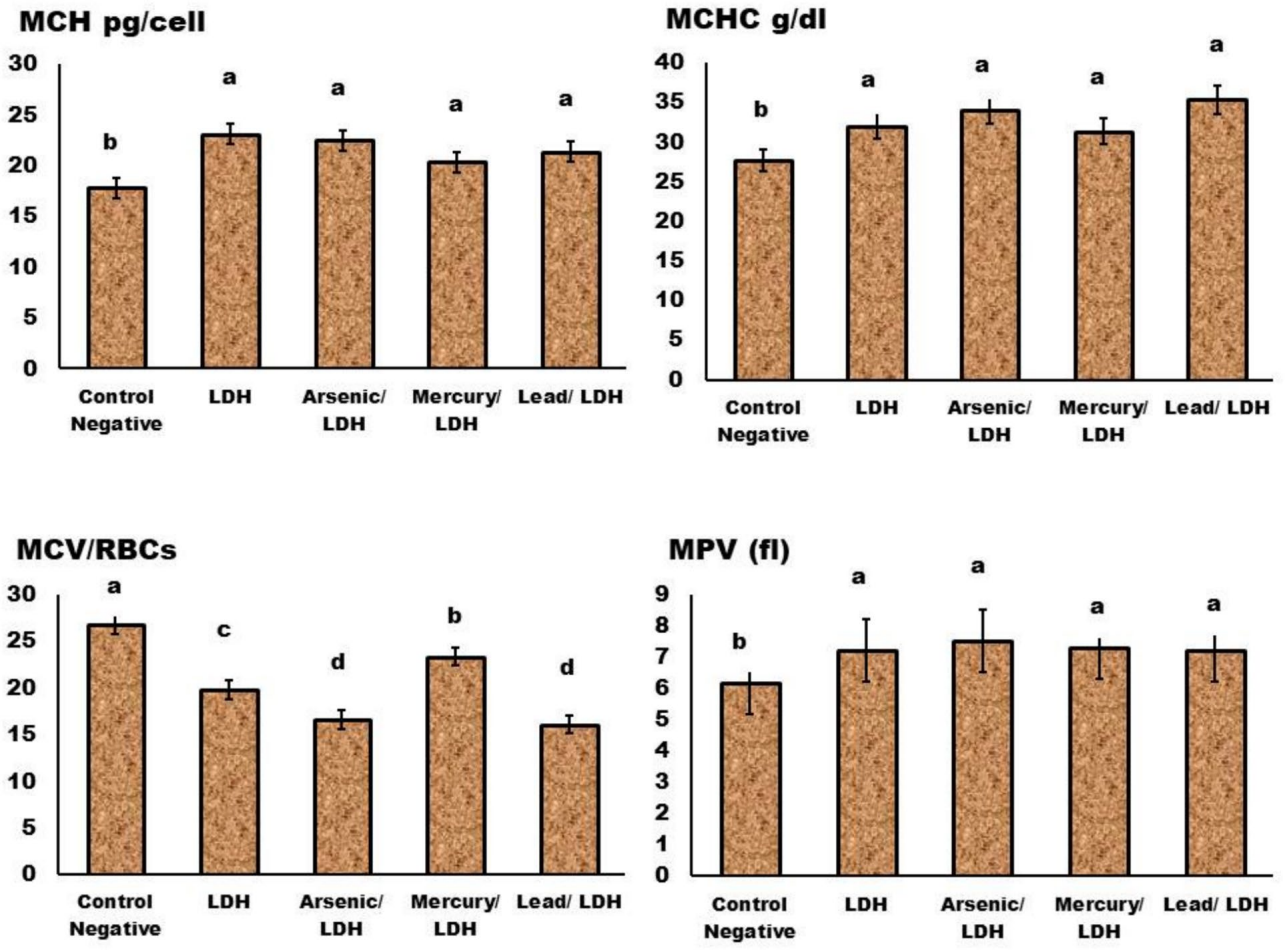
Effect of LDH, alone or after loading with As, Hg and pb on MCH, MCHC, MCV/RNCs, & MPV, no significant differences observed in between different groups with p value > 0.05 represented by same symbol whereas different superscript indicate significant difference than control negative *p* < *0.05.* mean ± SD (n = 6 per group). Significant differences compared to the control are indicated by letters as determined by One-Way ANOVA followed by Tukey’s post-hoc test.

**Fig. 13:**
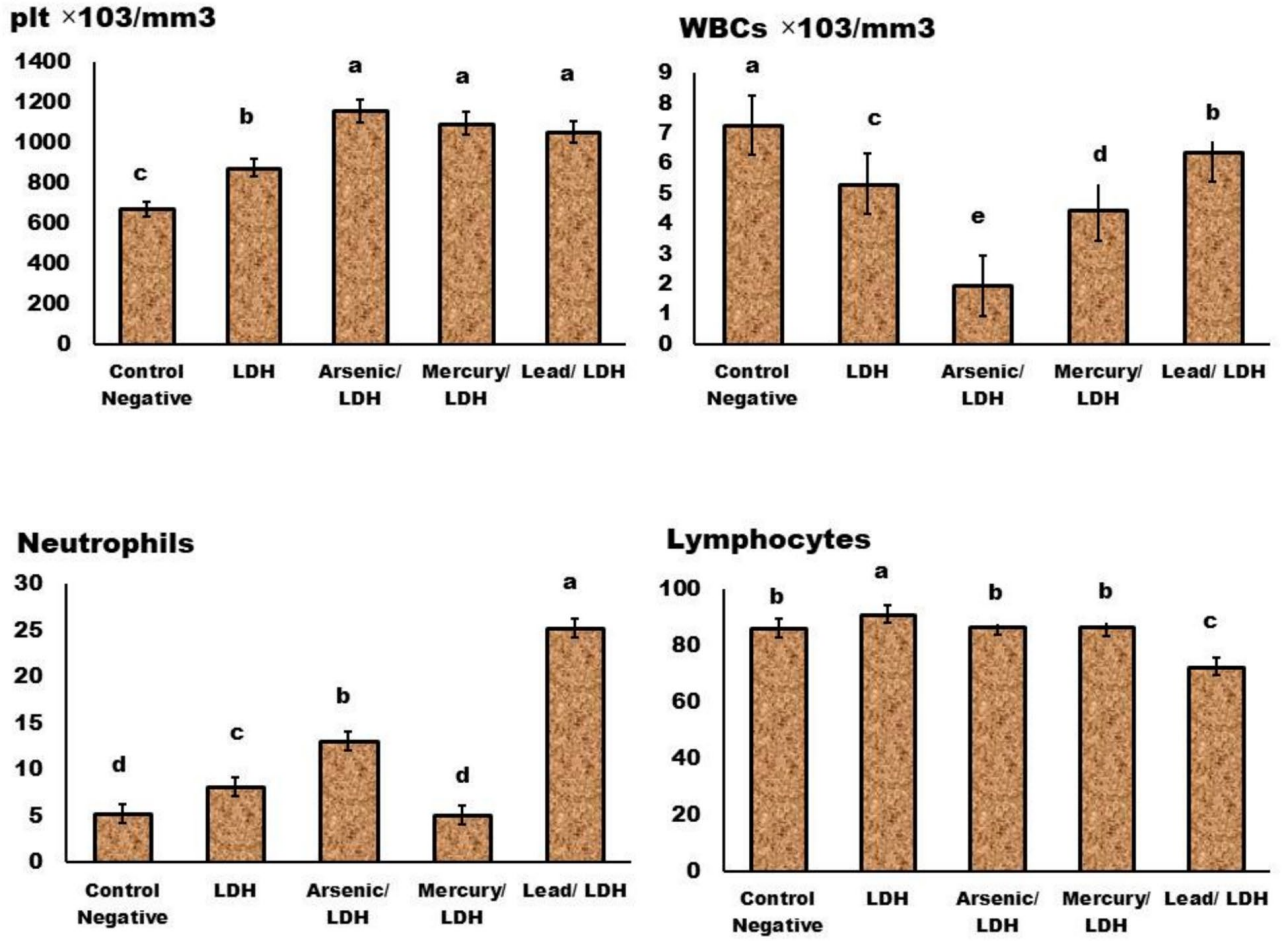
Effect of LDH, alone or after loading with As, Hg and pb on Plt, WBCs, Neutrophils and lymphocytes, no significant differences observed in between different groups with p value > 0.05 represented by same symbol whereas different superscript indicate significant difference than control negative *p* < *0.05.* mean ± SD (n = 6 per group). Significant differences compared to the control are indicated by letters as determined by One-Way ANOVA followed by Tukey’s post-hoc test.

**Fig. 14:**
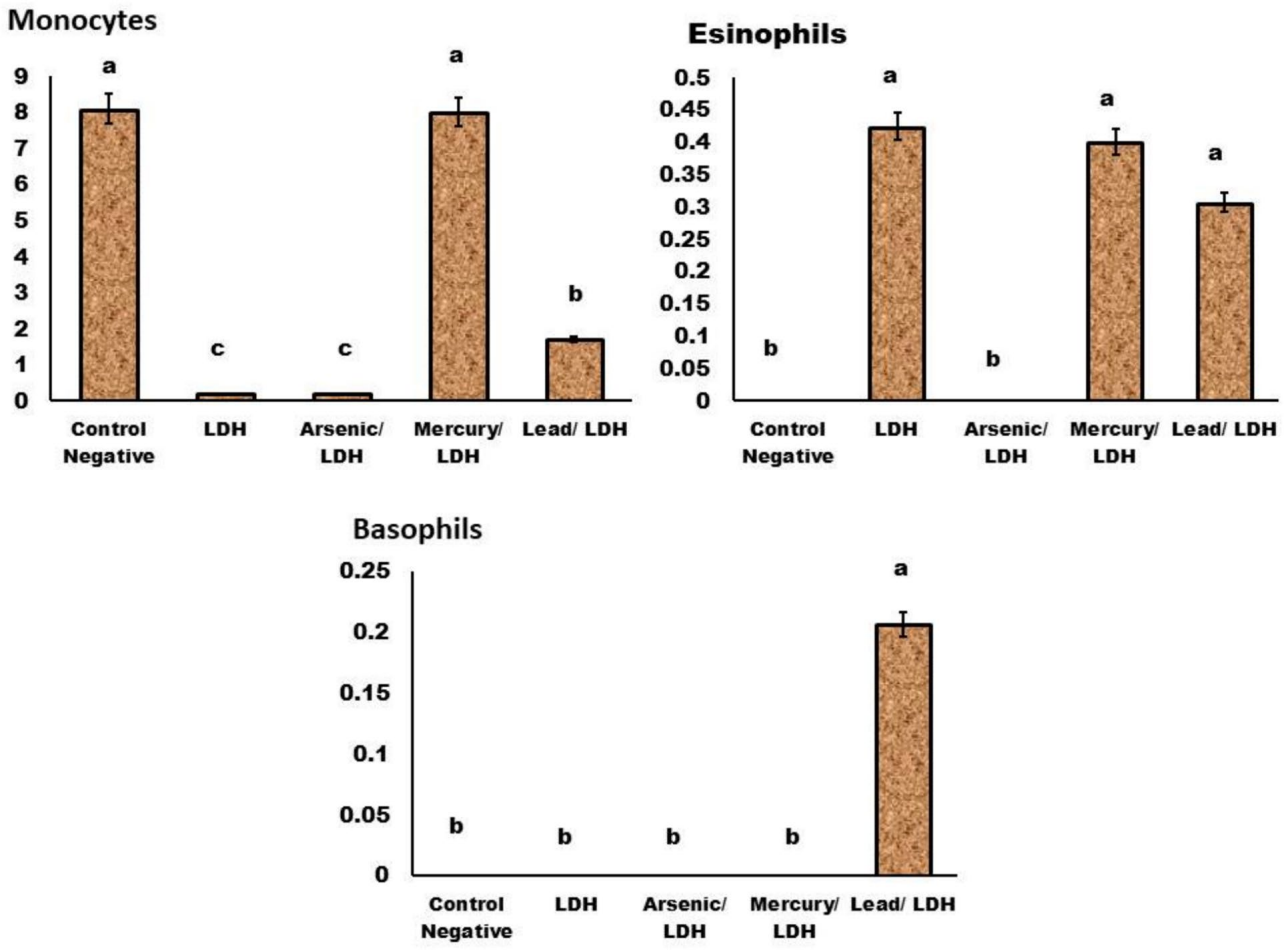
Effect of LDH, alone or after loading with As, Hg and pb on Monocytes, eosinophils and basophils, no significant differences observed in between different groups with p value > 0.05 represented by same symbol whereas different superscript indicate significant difference than control negative *p* < *0.05.* mean ± SD (n = 6 per group). Significant differences compared to the control are indicated by letters as determined by One-Way ANOVA followed by Tukey’s post-hoc test.

About the complete blood picture (CBC) (Figs. 11, 12, 13 and 14); in general normal blood picture without severely affected damage observed in blood parameters except some parameters increases or decreased according to different treatment. The eosinophilic cell count at LDH, LDH/Hg, and LDH/Pb increased significantly (P < 0.05). Your eosinophil concentrations in your blood can be elevated by a variety of conditions. Certain conditions, such as seasonal allergies and asthma, are exacerbated by the frequent adverse reactions to medications. Significant decrease in the monocytes count at *P* < *0.05* observed in LDH, LDH/pb and LDH/As admisntrations. Low levels of monocytes typically develop due to diseases or medical problem that reduce white blood cell count therapies that inhibit the immune system^70^. About the basophilic count significant increase when compared to control negative only in LDH/pb which indicate basophilia may indicate an infection or the presence of severe medical conditions such as leukaemia or autoimmune disease. Lead has a significant impact on the immune system, resulting in severe disorders. Basophils are a form of white blood cell that safeguards the body from infections.

While blood cells significantly *P* < *0.05* decrease in LDH, LDH/AS and LDH/pb occurs when the bone marrow fails to produce an adequate quantity of red and white blood cells, as well as platelets which means that’s its severely affected^70^. Platelet count significantly increased in LDH combined with either Hg, Pb and Arsenic which might indicate^71^ inflammatory conditions occurred specially in the digestive system. Neutrophilia increased, particularly in LDH/AS and LDH/pb, which suggests a severe inflammatory condition. The term “neutrophilia” refers to a condition in which the absolute neutrophil concentration in the blood exceeds the normal reference range. The presence of neutrophils may be indicative of infections, inflammation, and/or neoplastic processes^72^^,^^73^.

The LDHs that were generated prior to and following the adsorption process did not significantly alter the hemoglobin concentration or blood cell count in the rats. The efficacy of LDHs in mitigating biochemical changes was additionally confirmed through histological examinations of liver and kidney samples. These analyses demonstrated a decrease in toxicity levels as a result of the adsorption of highly hazardous metals, including lead, mercury, and arsenic, by LDH as previously documented in literature pertaining to the toxic effects of these metals on bodily functions and histological investigations^74^.

During a 14-day period, rats that were administered orally with LDHs exhibited minor hematological modifications, specifically affecting parameters such as hemoglobin (Hb) levels, red blood cell (RBC) count, and packed cell volume (PCV). All other hematological parameters exhibited statistically insignificant elevations in their respective counts. The study investigated the acute toxicity of LDH iron derived from hemoglobin of lysed erythrocytes in rats. It was observed that LDH iron has the potential to transform hydrogen peroxide (H_2_O_2_) into hydroxyl radicals, hence intensifying oxidative stress. One of the findings of our research is the reduction in blood hemoglobin levels and erythrocyte count, which is supported by the aforementioned arguments.

According to recent studies, the administration of LDH for the purpose of adsorbing heavy metals has been found to induce toxicity. Specifically, the exposure of various types of LDHs at doses comparable to those experienced by humans over extended periods of time has been observed to have an adverse impact on liver function. This is evidenced by elevated levels of SGPT (serum glutamate pyruvate transaminase) and SGOT (serum glutamate oxaloacetate transaminase), particularly. In the context of heavy metals poisoning, the presence of metabolically hazardous intermediates may induce structural impairments in the liver, hence altering the permeability of hepatocyte membranes for the transportation of chemicals.

The occurrence of liver enzyme leakage, particularly the release of SGPT and SGOT, has been extensively documented in instances of heavy metals intoxication. The observed elevation in levels of SGPT and SGOT in this study can perhaps be related to the leakage of hepatic enzymes. The decrease in alanine and aspartate transaminase activity in hepatic enzymes, as shown, suggests that heavy metals exert deleterious impacts on hepatic functionality. Nevertheless, it is important to acknowledge that the decline was partially counteracted by the process of LDH adsorption.

**Fig. 15:**
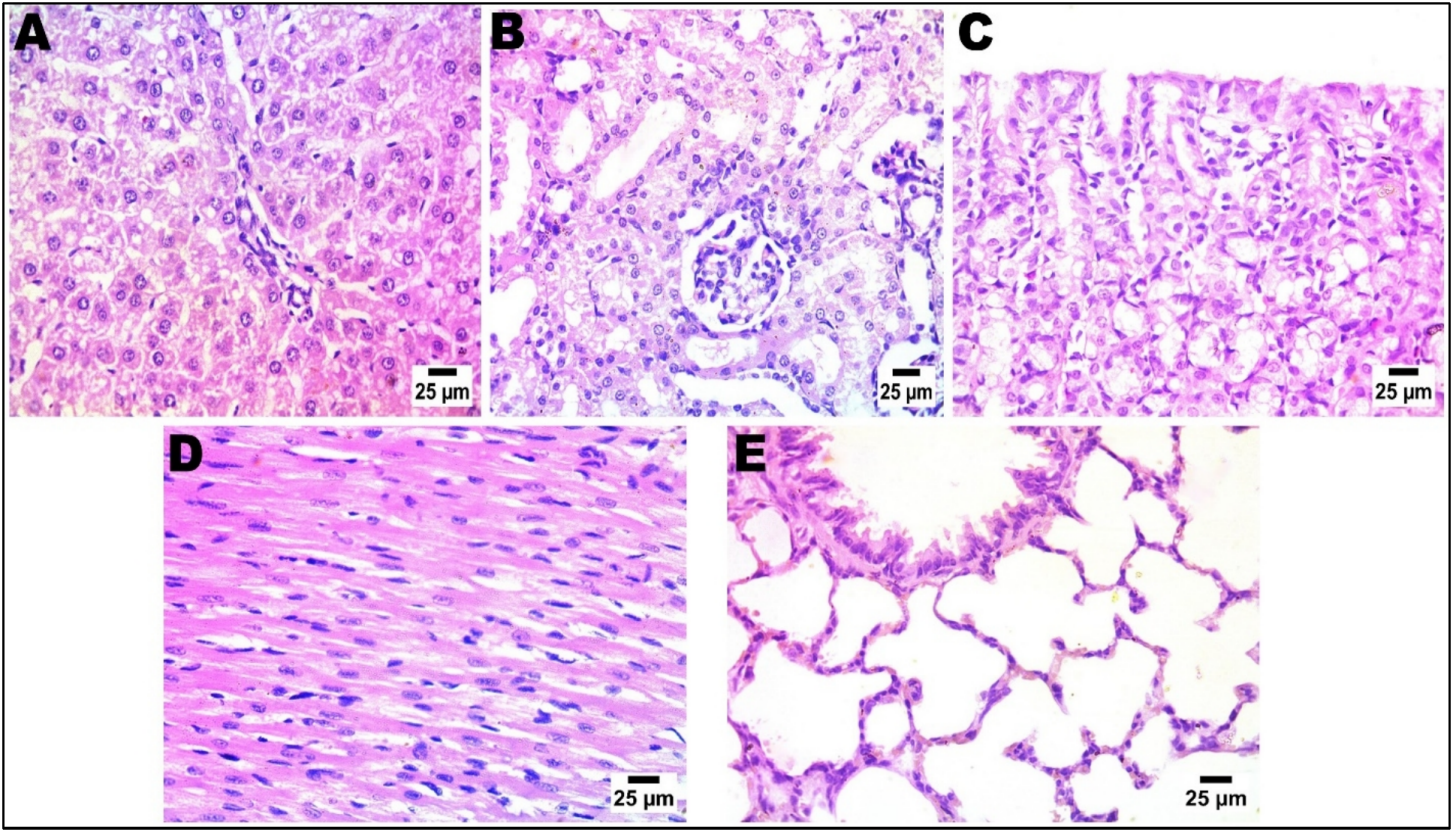
In control negative normal rats; photomicrograph in liver showing normal histological structure of portal area (**A**), The regular tissue arrangement of the gastric mucosa and the glomeruli and renal tubules (**B**) remain unchanged. (**C**), normal histological structure of cardiac muscle fibres (**D**), & normal histological structure of alveoli and bronchiole (**E**) (Haematoxylin and Eosin stain).

**Fig. 16:**
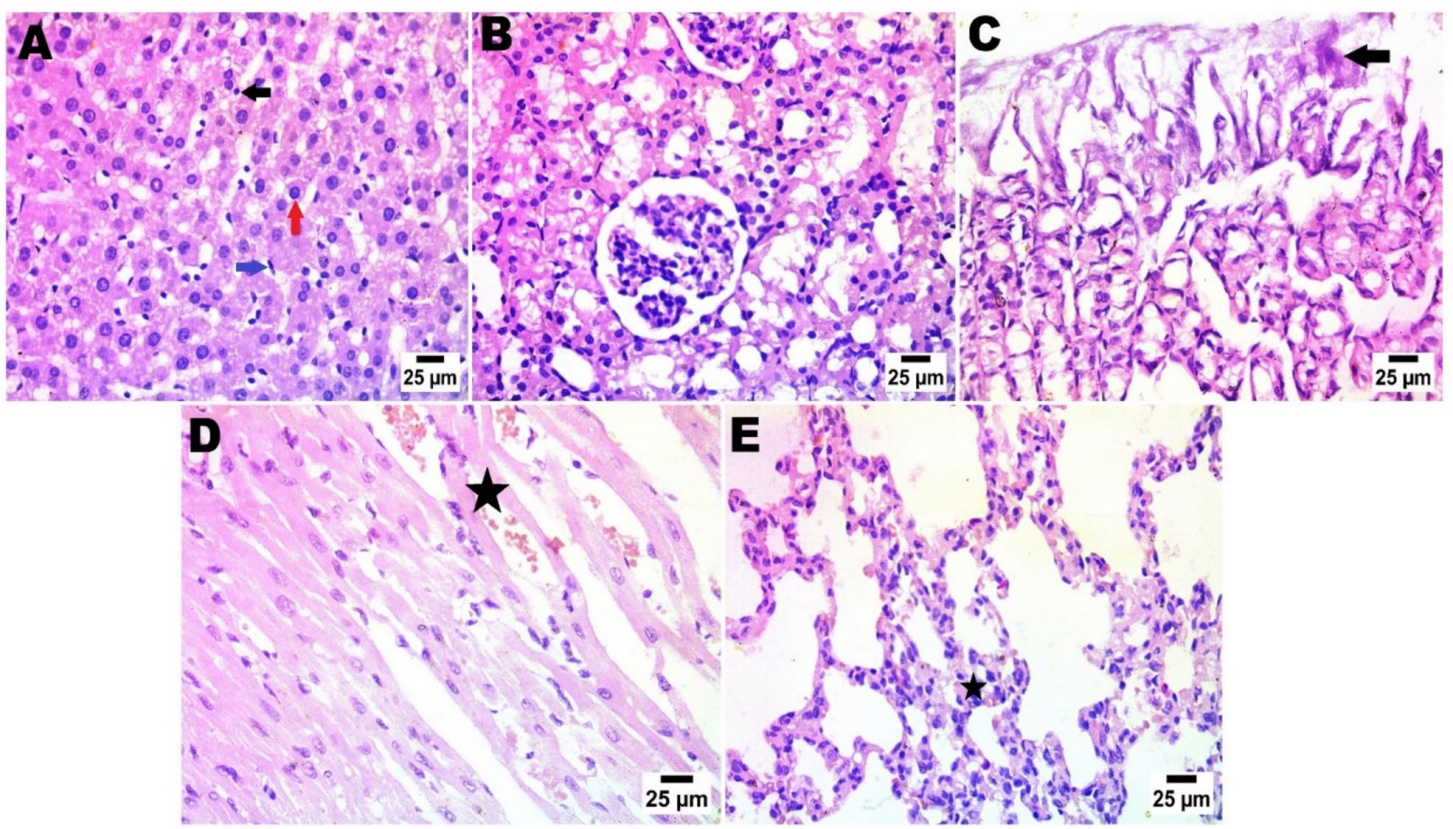
After arsenic adsorption photomicrograph showing nuclear pyknosis in hepatocytes (red arrow), presence of sinusoid dilatation with activation of van Kupffer cells (blue arrow) and few numbers of mononuclear inflammatory cells infiltration (black arrow) (**A**), the typical histological structure of the renal tubules and glomeruli (**B**), simple necrobiotic changes with sloughing of gastric epithelium (arrow) (**C**), disorganization and disarrangement of cardiac muscle fibres with haemorrhage (star) (**D**), & interstitial pneumonia by increasing thickness of alveolar wall and presence of inflammatory cells (star)(Haematoxylin and Eosin stain).

**Fig. 17:**
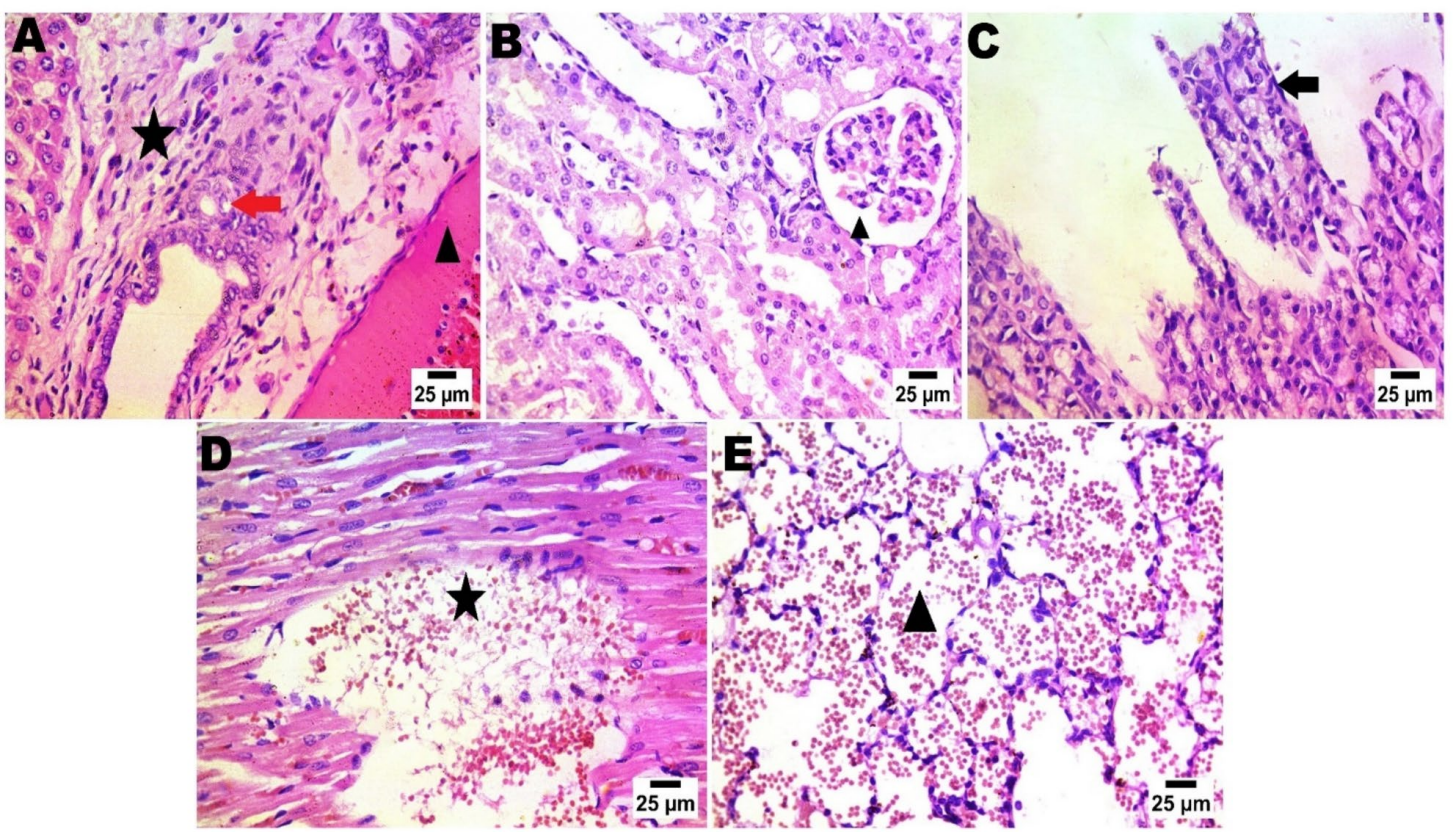
photomicrograph in Zn-Fe-LDH after lead adsorption showing portal fibrosis with inflammatory markers (star) and congestion of portal blood vessels (arrow head), presence of newly formed bile ductules (arrow) (**A**), increase in urinary space of glomeruli (**B**), severe sloughing of gastric epithelium and severe disarrangement besides disorganization of cardiac muscle fiber with haemorrhage (star) (**D**), & sever alveolar haemorrhage in lung alveoli (**E**). (Haematoxylin (**C**), and Eosin stain).

**Fig. 18:**
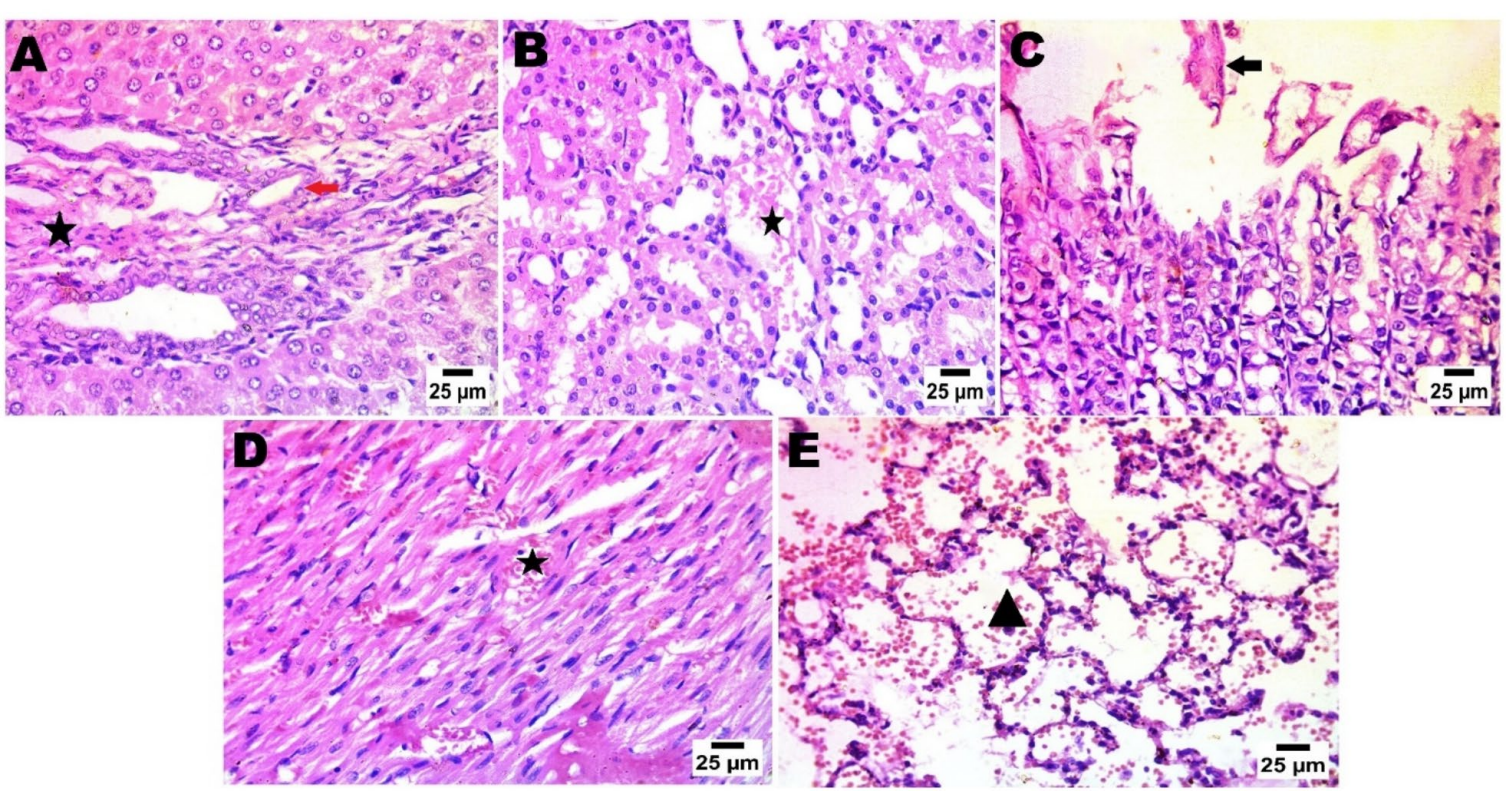
After mercury adsorption by LDH; photomicrograph showing portal fibrosis with infiltration by mononuclear inflammatory cells (star) and presence of newly formed bile ductules (arrow) (**A**), pre-tubular blood vessel congestion (**B**), sloughing of gastric epithelium (arrow) (**C**), in heart; showing disorganization of cardiac muscle fibres with haemorrhage (star) (**D**), & sever alveolar haemorrhage in lung (**E**). (Haematoxylin and Eosin stain).

**Fig. 19:**
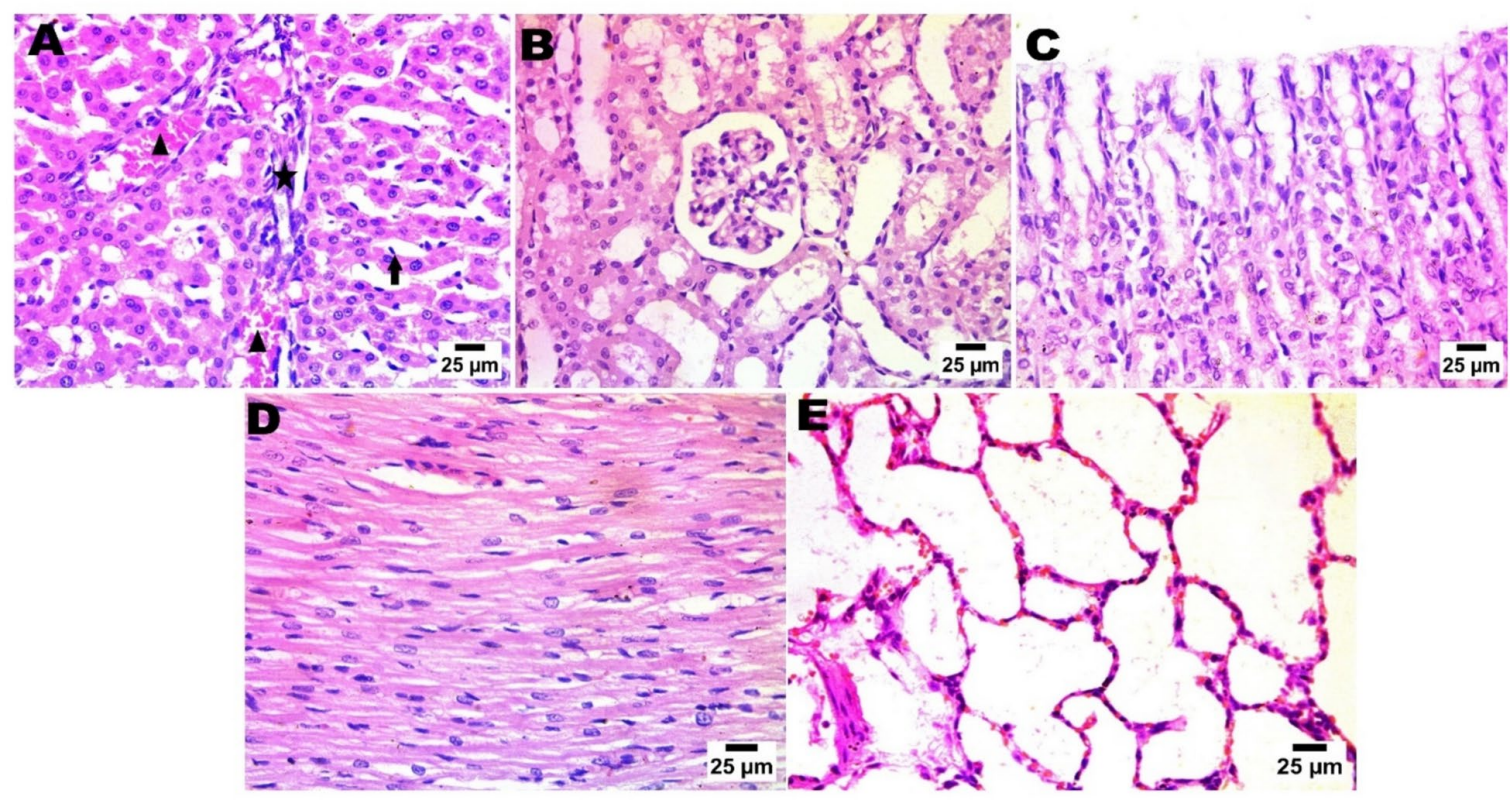
LDH before adsorption (only LDH without chelation), in liver photomicrograph showing formation of connective tissue bridge (star) with congested portal blood vessels (arrow head), presence of sinusoid dilatation with activation of van Kupffer cells (arrow) (**A**), normal histological structure of glomeruli and renal tubules (**B**), normal histological structure of gastric mucosa (**C**), normal histological structure of cardiac muscle fibres in heart tissue (**D**), & normal lung alveoli and tissue (Haematoxylin and Eosin stain).

As depicted in Fig. 15 (normal control group), Fig. 16 (LDH/AS), Fig. 17 (LDH/Pb), Fig. 18 (LDH/Hg), and Fig. 19 (LDH only), the administration of several types of, LDHs In adult male albino rats from the study groups, histological images of hepatic tissues showed a normal central vein (C), a normal arrangement of hepatocytes forming plates separated by hepatic sinusoids (arrowheads), and normal portal areas with intact arteries. before and after adsorption does not result in significant changes in the histological appearance or any injury to the liver, kidney, or other organs. The histological structure of the liver was typical, as evidenced by Histological images of hepatic tissues in adult male albino rats from the study groups the presence of a normal central vein (C), a normal arrangement of hepatocytes forming plates that are separated by hepatic sinusoids (shown by arrowheads), and normal portal areas containing intact arteries (P).. The LDH alone group had histological features consistent with normal liver structure, including intact portal areas (P), central veins (C), and hepatocytes. Upon examination, it was seen that LDH exhibited a rather typical appearance, characterized by a central vein (C) that appeared normal. However, the portal veins displayed slight congestion. The majority of hepatocytes had normal morphology, but a small number of cells displayed mild degeneration in the vicinity of the portal area, as depicted in the histological images.

The renal histological architecture was within the normal range in the sections from the control group. This encompassed the presence of intact renal tubules and normal renal corpuscles. The histological characteristics of the kidney in the LDH group were consistent with those of normal renal corpuscles and renal tubules. The renal tubules demonstrated significant deterioration and cystic dilatation in individuals with LDH/AS. Signs of congestion were observed in the renal blood vessels subsequent to lead absorption.

A range of organs were gathered for histological assessment, and further examination revealed no pathological findings in any of the organs following a 14-day acute treatment regimen. The hepatocytes, cardiomyocytes, stomach, alveolar, and renal organs exhibited regular arrangement. In the liver organ, the hepatic central vein and hepatic cords exhibit a high degree of patency. The kidney exhibited a typical arrangement of glomeruli, without any signs of congestion or cyanosis. The control negative and LDH samples exhibited typical histological structure prior to adsorption. The presence of lead and mercury during adsorption has been reported to result in damage and degenerative lesions in LDH. Conversely, the adsorption of arsenic has been found to cause the least amount of damage in LDH.

Medication delivery systems are becoming more common to improve therapeutic effectiveness, active or passive targeting, controlled or delayed release, and systemic drug side effects^75,76^. The increased interest in using layered double hydroxides (LDHs) as drug delivery vehicles is due to their biocompatibility, biodegradability, large storage capacity, and ability to promote modified release of immobilized species. Chemical composition, layer charge density, surface characteristics, and particle size appear to be connected to the performance of LDHs in interaction with biological systems, as measured by in vitro or in vivo experiments. When compared to pharmaceutical traditional systems, synthetic factors can adjust drug release in terms of rate, time, and/or release targeted^77^.

A new paradigm for medicinal administration in the field of pharmacology has garnered significant traction due to the safety and low toxicity of Zn-Fe-LDH. The acute toxicity of Zn-Co-Fe/LDH, Zn-Co-Fe/LDH-As, Pb, and Hg was assessed using the LD_50_ method in the acute toxicity study, as well as histopathological investigations of various body organs. LDH is often used as a nanomaterial carrier for proteins, genes, and medication delivery systems^78^.

Nanotechnology can manage and release drugs, helping treat a variety of biological problems. The Nano-Meter scale has allowed the creation of novel materials for cutting-edge medical technology and a comeback in multifunctional Nano carrier targeting effectiveness. By adding tiny molecules to nanoparticles or layers, drugs may be made safer, bioavailable, and more effective^79^. The dimension of the nano carrier and its integration into layers^79^ The nano carrier’s size and layer integration affect medication pharmacokinetics and pharmacodynamics^80^. Nanoparticles have a high surface-to-volume ratio, which enhances drug delivery. LDH layers are also excellent^81^. Enhances the efficacy and longevity of several drugs In ROS, LDH’s Zn and Fe ions are antibacterial and free radical scavengers. ROS production rises with oxidative stress, speeding up cellular activity. Zn and Fe help achieve high activity in little time^82^.Nanoparticle-containing drugs are effective in treating brain diseases and infections due to their small size particles adhering efficiently and passing through the blood–brain barrier, as well as their sustained or controlled release, which reduces dosing and drug side effects on organ function^83^.

To evaluate the acute oral toxicity of 100 nm LDH nanoparticles, rats were given four doses (5, 50, 300, and 2000 mg/kg). The study found that LDH nanoparticles did not cause any mortality, abnormal behaviors, symptoms, or weight loss at the maximal dosage, with LD_50_ values above 2000 mg/kg. LDH nanoparticles had no effect on organosomatic indices of any organs in rat, proving their minimal oral toxicity. The lack of a significant serum biochemical rise suggests LDH nanoparticles did not harm the liver or kidneys. Pharmacokinetic and tissue distribution studies showed that LDH nanoparticles were swallowed within 30 min and eliminated after 3.1 h, without accumulating in target tissues at a dosage of 2000 mg/kg. LDH nanoparticles were eliminated in urine and feces within 24 h. Conclusion: LDH nanoparticles do not cause acute oral toxicity in animals. Their quick systemic clearance and minimal gastrointestinal absorption are linked to this^84^. There are many previous literature confirmed the LDH safety on different body function and organs which confirmed our investigation at this study^85–88^.

The variation in LD_50_ values among the metal-laden adsorbents suggests a significant influence of the specific metal species and its binding affinity within the LDH framework. Zn-Co-Fe/LDH-As exhibited the lowest toxicity (LD_50_ = 370 mg/kg), which may be attributed to the strong oxo-anionic coordination of As^3+^ with the hydroxyl groups of the LDH brucite-like layers, as suggested by the [FTIR] results. This strong chemical interaction likely limits the bioavailability of arsenic in the gastrointestinal tract of the rats, reducing its systemic absorption. In contrast, the higher toxicity observed for LDH-Pb and LDH-Hg LD_50_ values of 103.7 and 204 mg/kg, respectively) may stem from the inherently higher mammalian toxicity of these cations and a potentially weaker surface-adsorption mechanism, allowing for greater metal dissociation and subsequent interference with cellular organelles and the endothelial glycocalyx^6^.

The observed toxicity was not a result of the chelation process itself, but rather a function of the metal loading on the Zn-Co-Fe/LDH framework. Specifically, the lower LD_50_ values for LDH-Pb and LDH-Hg indicate that the toxic potential is driven by the specific heavy metal sequestered within the matrix. While the LDH acts as a carrier, the inherent toxicity of the adsorbed Lead and Mercury species and their potential dissociation under gastric pH conditions-contributes to the biological response. This clarifies that while LDH effectively sequesters these metals from wastewater, the resulting spent adsorbent must be handled according to its specific metal-induced toxicity profile.

The histopathological alterations observed in the liver and kidneys reflect the distinct toxicodynamic pathways of the sequestered metals. In the LDH-Pb treated groups, sinusoid dilatation and portal fibrosis were prominent; these are classic indicators of lead-induced oxidative stress, which triggers the activation of hepatic stellate cells and subsequent collagen deposition.

In contrast, the LDH-Hg groups exhibited significant renal tubular necrosis and glomerular shrinkage, likely due to mercury’s high affinity for sulfhydryl groups in mitochondrial proteins, leading to acute cellular energy failure and apoptosis. The relatively milder portal inflammation seen in LDH-As groups correlates with its higher LD50, suggesting that the strong coordination of As^3+^ within the LDH layers (as confirmed by FTIR) successfully limited the induction of reactive oxygen species (ROS) compared to the more labile Pb and Hg complexes.

## Conclusion

This study provides a comprehensive toxicological validation of Zn-Co-Fe/LDH as a robust framework for heavy metal sequestration. By employing Probit analysis, we successfully quantified the safety margins for both pristine and metal-laden adsorbents, establishing a clear toxicity hierarchy of As < Hg < Pb. The results demonstrate that while the pristine LDH possesses an excellent safety profile, the ‘spent’ adsorbents carrying Lead and Mercury induce significant inflammatory responses, evidenced by neutrophilia and altered hepatic enzyme activities. In contrast, the LDH-Arsenic complex exhibited a markedly higher LD_50_(370 mg/kg) and maintained biochemical parameters (urea, creatinine, and troponin) near control levels, suggesting superior chemical stabilization of Arsenic within the LDH layers. These findings transition the study from simple adsorption efficiency to a ‘cradle-to-grave’ safety assessment, providing the necessary LD_50_ benchmarks and 1/20th safety thresholds required for the responsible industrial handling, valorization, and disposal of spent LDH adsorbents in wastewater management systems. Toxicological evaluation using Probit analysis successfully established the safety profiles of Zn-Co-Fe/LDH before and after heavy metal sequestration. Pristine LDH demonstrated the highest safety margin, while the metal-laden ‘spent’ adsorbents exhibited a toxicity hierarchy of As < Hg < Pb. Specifically, LDH-As showed the lowest biological impact (LD_50_ = 370 mg/kg) compared to the more severe toxic symptoms and lower LD_50_ values associated with Pb and Hg. These results define the sub-lethal safety benchmarks required for the responsible handling and disposal of metal-laden adsorbents in industrial waste management.

### Novelty of the study

This investigation provides a quantitative toxicological profile of Zn-Co-Fe/LDH before and after the adsorption of heavy metals (As3 + , Pb2 + , and Hg2 +). The use of Probit Analysis successfully established a toxicity hierarchy, identifying LDH-As (LD50 = 370 mg/kg) as significantly less toxic than LDH-Pb and LDH-Hg. While the pristine LDH demonstrates a high safety margin, the metal-laden ‘spent’ adsorbents exhibit dose-dependent histopathological changes in the liver and kidneys. These findings establish critical safety benchmarks (1/20th of LD50) for the handling and sustainable disposal of these materials in industrial wastewater treatment frameworks.

The novelty of this work lies in the multi-dimensional optimization of the Zn-Co-Fe/LDH adsorbent. In terms of cost-effectiveness, the LDH framework utilizes abundant transition metals, providing a low-cost alternative to expensive commercial chelators. Regarding reliability, the application of Probit Analysis provides a statistically robust and reproducible mathematical model for predicting LD_50_, moving beyond qualitative safety estimates. Finally, the performance novelty is demonstrated by the material’s ability to not only sequester heavy metals but to chemically stabilize them, significantly reducing their bioavailability and systemic toxicity-as evidenced by the high LD_50_ values compared to free metal salts. This establishes a ‘safety-by-design’ approach for wastewater treatment materials.

### Future perspectives

Building upon the established acute safety profile of Zn-Co-Fe/LDH, future research should focus on chronic and sub-chronic toxicity studies to evaluate the potential for bioaccumulation over extended exposure periods. Additionally, leaching tests under varying environmental conditions (such as fluctuating soil pH or microbial activity) are necessary to ensure the long-term stability of the sequestered metals in landfill scenarios. From an engineering perspective, the regeneration and reuse cycles of the LDH should be investigated to determine how multiple adsorption–desorption cycles affect the material’s structural integrity and toxicological footprint. Finally, transitioning from laboratory rat models to ecotoxicological assays involving aquatic organisms (e.g., *Daphnia magna*) would provide a more holistic understanding of the environmental safety of these adsorbents during large-scale industrial wastewater applications.

## Supplementary Information

Supplementary Information.

## Data Availability

The datasets used and/or analysed during the current study available from the corresponding author on reasonable request.

## Abbreviations

As^3+^: Arsenic
EDX: Energy-dispersive X-ray spectroscopy
FTIR: Fourier transform infra red
Hg^2+^: Mercury
LD50: The amount of a substance such as a drug, chemical, or poison—required to kill 50% of a test population (usually mice or rats) in a single exposure
LD100: The lowest amount of a substance, poison, or radiation that causes death in 100% of a test population
LDH: Layered double hydroxide
Pb^2+^: Lead
SEM: Scanning electron microscope
XRD: X-Ray diffraction

## References

[1] Ghorani-Azam, A., Riahi-Zanjani, B. & Balali-Mood, M. Effects of air pollution on human health and practical measures for prevention in Iran. *J. Res. Med. Sci.* **21**, 65. 10.4103/1735-1995.189646 (2016). 27904610 10.4103/1735-1995.189646 PMC5122104

[2] Luo, L. et al. Heavy metal contaminations in herbal medicines: determination. comprehensive risk assessments. *Front. Pharmacol.* **11**, 595335. 10.3389/fphar.2020.595335 (2020). 33597875 10.3389/fphar.2020.595335 PMC7883644

[3] Mousavi, S. R., Balali-Mood, M., Riahi-Zanjani, B., Yousefzadeh, H. & Sadeghi, M. Concentrations of mercury, lead, chromium, cadmium, arsenic and aluminum in irrigation water wells and wastewaters used for agriculture in Mashhad, northeastern Iran. *Int. J. Occup. Environ. Med.* **4**(2 April), 80–86 (2013). 23567533

[4] Kotnala, S. et al. Impact of heavy metal toxicity on the human health and environment. *Sci. Total Environ.* **987**, 179785. 10.1016/j.scitotenv.2025.179785 (2025). 40466229 10.1016/j.scitotenv.2025.179785

[5] Wang, S. & Shi, X. Molecular mechanisms of metal toxicity and carcinogenesis. *Mol. Cell. Biochem.* **222**, 3–9 (2001). 11678608

[6] Kang, H. et al. The substructure of the endothelial glycocalyx in rat aorta. *Am. J. Pathol.* **195**(10), 1936–1958 (2025). 40645580 10.1016/j.ajpath.2025.06.005 PMC12597546

[7] Gupta, D. K., Tiwari, S., Razafindrabe, B. & Chatterjee, S. Arsenic contamination from historical aspects to the present. In *Arsenic Contamination in the Environment* 1–12 (Springer, 2017). 10.1007/978-3-319-54356-7_1.

[8] Mochizuki, H. Arsenic neurotoxicity in humans. *Int. J. Mol. Sci.* **20**(14), 3418. 10.3390/ijms20143418 (2019). 31336801 10.3390/ijms20143418 PMC6678206

[9] Ragab, A. H., Gumaah, N. F., Elfiky, A. A. & Mubark, M. F. Exploring the sustainable elimination of dye using cellulose nanofbrils- vinyl resin based nanofltration membranes. *BMC Chem.* **18**, 121 (2024). 38937828 10.1186/s13065-024-01211-5 PMC11212259

[10] Sattar, A. et al. Metabolism and toxicity of arsenicals in mammals. *Environ. Toxicol. Pharmacol.* **48**, 214–224. 10.1016/j.etap.2016.10.020 (2016). 27829199 10.1016/j.etap.2016.10.020

[11] Shah, A. Q. et al. Determination of inorganic arsenic species (As3+ and As5+) in muscle tissues of fish species by electrothermal atomic absorption spectrometry (ETAAS). *Food Chem.* **119**(2), 840–844. 10.1016/j.foodchem.2009.08.041 (2010).

[12] Jacobs, D. E., Wilson, J., Dixon, S. L., Smith, J. & Evens, A. The relationship of housing and population health: a 30-year retrospective analysis. *Environ. Health Perspect.* **117**(4), 597–604. 10.1289/ehp.0800086 (2009). 19440499 10.1289/ehp.0800086 PMC2679604

[13] Joseph, C. L. M. et al. Blood lead level and riskofasthma. *Environ. Health Perspect.* **113**(7), 900–904. 10.1289/ehp.7453 (2005). 16002380 10.1289/ehp.7453 PMC1257653

[14] Kianoush, S. et al. Comparison of the rapeutic effects of garlic and d-penicillamine in patients with chronic occupational lead poisoning. *Basic Clin. Pharmacol. Toxicol.* **110**(5), 476–481 (2012). 22151785 10.1111/j.1742-7843.2011.00841.x

[15] Kasten-Jolly, J., Heo, Y. & Lawrence, D. A. Impact of developmental lead exposure on splenic factors. *Toxicol. Appl. Pharmacol.* **247**(2), 105–115 (2010). 20542052 10.1016/j.taap.2010.06.003 PMC3219921

[16] Hemdan, M. et al. Sustainable synthesis and environmental application of chitosan-Ocimum basilicum leaves-ZnO composite membrane for permanganate ion removal in wastewater treatment. *Environ. Sci. Pollut. Res.* **31**, 66164–66183 (2024). 10.1007/s11356-024-35612-9 39621216

[17] Mishra, K. P. Lead exposure and its impact on immune system: a review. *Toxicol. Vitr.* **23**(6), 969–972 (2009). 10.1016/j.tiv.2009.06.014 19540334

[18] Burki, T. K. Nigeria’sleadpoisoningcrisiscould leavea long legacy. *Lancet* **379**(9818), 792. 10.1016/s0140-6736(12)60332-8 (2012). 22393573 10.1016/s0140-6736(12)60332-8

[19] Kianoush, S. et al. Clinical toxicological biochemical and hematologic parameters in lead exposed workers of a car battery industry. *Iran J. Med. Sci.* **38**(1), 30 (2013). 23645955 PMC3642942

[20] Kianoush, S., Sadeghi, M. & Balali-Mood, M. Recent advances in the clinical management of lead poisoning. *Acta Med. Iran* **53**, 327–336 (2015). 26069169

[21] Devlin, E. W. Acute toxicity, uptake and histopathology of aqueous methyl mercury to fathead minnow embryos. *Ecotoxicology* **15**, 97–110. 10.1007/s10646-005-0051-3 (2006). 16400529 10.1007/s10646-005-0051-3

[22] Pack, E. C. et al. Effects of environmental temperature change on mercury absorption in aquatic organisms with respect to climate warming. *J. Toxicol. Environ. Health A* **77**, 1477–1490. 10.1080/15287394.2014.955892 (2014). 25343296 10.1080/15287394.2014.955892

[23] Bakir, F. et al. Methylmercury poisoning in Iraq. *Science* **181**, 230–241 (1973). 4719063 10.1126/science.181.4096.230

[24] TsubAki, T. K. & Irukayama, K. *Minamata Disease: Methyl Mercury Poisoning in Minamata and Niigata, Japan* 8–34 (Elsevier Scientific Publ Co, 1977).

[25] Pelalak, R., Hassani, A., Heidari, Z. & Zhou, M. State-of-the-art recent applications of layered double hydroxides (LDHs) material in Fenton-based oxidation processes for water and wastewater treatment. *Chem. Eng. J.* **474**, 145511 (2023).

[26] Mubarak, M. F., Selim, H., Hawash, H. B. & Hemdan, M. Flexible, durable, and anti-fouling maghemite copper oxide nanocomposite-based membrane with ultra-high fux and efciency for oil-in-water emulsions separation. *Environ. Sci. Pollut. Res. Int.* **31**, 2297–2313 (2024). 38062214 10.1007/s11356-023-31240-x PMC10791961

[27] Mubarak, M. F., Khedr, G. E. & El Sharkawy, H. M. Corrigendum to" environmentally-friendly calcite scale mitigation: encapsulation of CDs@ MS composite within membranes framework for nanofiltration". *J. Alloys Compd.* **1000**, 175151 (2024).

[28] Rajamathi, M., Thomas, G. S. & Kamath, P. V. The many ways of making anionic clays. *J. Chem. Sci.* **113**, 671–680 (2001).

[29] Mahmoud, R. et al. Novel anti-inflammatory and wound healing controlled released LDH-Curcumin nano composite via intramuscular implantation, in-vivo study. *Arab. J. Chem.* **15**, 103646. 10.1016/j.arabjc.2021.103646 (2022).

[30] El- Sawaf, A. K. et al. Green synthesis and characterization of magnetic gamma alumina nanoparticlesfor copper ions adsorption from synthetic wastewater. *Results Eng.* **22**, 101971 (2024).

[31] Mahmoud, R. K., Taha, M., Zaher, A. & Amin, R. M. Understanding the physicochemical properties of Zn–Fe LDH nanostructure as sorbent material for removing of anionic and cationic dyes mixture. *Sci. Rep.* **11**, 21365. 10.1038/s41598-021-00437-w (2021). 34725383 10.1038/s41598-021-00437-w PMC8560778

[32] Wang, W., Wang, S., Ma, X. & Gong, J. Recent advances in catalytic hydrogenation of carbon dioxide. *Chem. Soc. Rev.* **40**(7), 3703–3727 (2011). 21505692 10.1039/c1cs15008a

[33] Zhang, X. et al. Using biochar for remediation of soils contaminated with heavy metals and organic pollutants. *Environ. Sci. Pollut. Res.* **20**, 8472–8483 (2013). 10.1007/s11356-013-1659-0 23589248

[34] Smith, J. A., Rahman, M. L. & Chen, Y. Advanced layered double hydroxides (LDHs) for the efficient removal of arsenic, lead, and mercury from industrial wastewater: mechanisms and adsorption kinetics. *J. Environ. Chem. Eng.* **11**(3), 109–125 (2023).

[35] Aita, S. A., Mahmoud, R., Hafez, S. H. & Zaher, A. Investigating adsorption of aqueous heavy metals through isotherms and kinetics with Zn-Co-Fe/LDH for remarkable removal efficiency. *Appl. Water Sci.* **15**(4), 75 (2025).

[36] Organization for Economic Co-operation and Development (OECD). *Guidance document on acute oral toxicity testing 420* (Organization for Economic Co-operation and Development, 2008).

[37] World Health Organization (WHO). General Guidelines for methodologies on research and evaluation of traditional medicine; WHO: Geneva. *Switzerland* **2000**, 35 (2000).

[38] Das, N., Goshwami, D., Hasan, M., Sharif, R. & Zahir, S. Evaluation of acute and subacute toxicity induced by methanol extract of *Terminalia citrina* leaves in Sprague Dawley rats. *J. Acute Dis.* **4**, 316–321 (2015).

[39] El- Sawaf, A. K., Nassar, A. A., El Fiky, A. A. & Mubarak, F. M. Advanced microcrystalline nanocellulose-based nanofiltration membranes for the efficient treatment of wastewater contaminated with cationic dyes. *Polym. Bull.* **81**(14), 12451–12476 (2024).

[40] Finney, D. J. *Probit Analysis* 3 ed. (University Press, 1971).

[41] Miller, C. L. & Tainter, M. L. Estimation of LD50 and its error by means of log-probit graph paper. *Proc. Soc. Exp. Biol. Med.* **57**, 261–264 (1944).

[42] Arambaši, M. B., Kondi, S., Piti, L. J. & Stojanovi, M. Review of some mathematical statistical methods for processing toxicological- pharmacological experimental results. *Acta Pharm. Jugosl.* **41**(3), 177–190 (1991).

[43] Randhawa, M. A. Calculation of LD50 values from the method of Miller and Tainter, 1944. *J. Ayub Med. Coll. Abbottabad.* **21**(3), 184–185 (2009). 20929045

[44] Petz, B. Razlika izmeðu dva koeficijenta korelacije r. In *Osnovne statisti ke metode za. nematemati are* 218–219 (SNL, 1981).

[45] Mobolaji, J., Muhammad, H. L., Makun, A. H., Busari, B. M. & Abdullah, S. A. Acute and subacute toxicity studies of aqueous and methanol extracts of *Nelsonia campestris* in rats. *J. Acute Dis.* **5**, 62–70 (2016).

[46] Yuet, P.K., Darah, I., Chen, Y., Sreeramanan, S., & Sasidharan, S. Acute and subchronic toxicity study of Euphorbia hirta L. methanol extract in rats. *Biomed. Res. Int.* 182064–182071 (2013). 10.1155/2013/182064 PMC3872372 24386634

[47] Bigoniya, P., Sahu, T. & Tiwari, V. Hematological and biochemical effects of sub-chronic artesunate exposure in rats. *Toxicol. Rep.* **2**, 280–288 (2015). 28962361 10.1016/j.toxrep.2015.01.007 PMC5598518

[48] Dacie, S. J. V. & Lewis, S. M. *Practical Hematology* 24–45 (Churchill Livingstone, 1984).

[49] Ghule, B. V., Murugananthan, G., Nakhat, P. D. & Yeole, P. G. Immunostimulant effects of *Capparis zeylanica* Linn. leaves. *J. Ethnopharmacol.* **108**(2), 311–315 (2006). 16766150 10.1016/j.jep.2006.03.041

[50] Bancroft, J. D. *Theory and Practice of Histological Techniques* 6th Edn. (Elsevier Heal Sci., 2008).

[51] Snedecor, G. W. C., & William, G. Statistical methods/george w, Snedecor William g. Cochran. (1989).

[52] Ma, L. et al. Highly selective and efficient removal of heavy metals by layered double hydroxide intercalated with the MoS_4_^2-^ ion. *J. Am. Chem. Soc.* **138**, 2858–2866 (2016). 26829617 10.1021/jacs.6b00110

[53] Zhang, H., Li, G., Deng, L., Zeng, H. & Z. Shi, Z. Heterogeneous activation of hydrogen peroxide by cysteine intercalated layered double hydroxide for degradation of organic pollutants: Performance and mechanism. *J. Colloid Interface Sci.* **543**, 183–191 (2019). 30802765 10.1016/j.jcis.2019.02.059

[54] Zhang, X., Yan, L., Li, J., & Yu, H. Adsorption of heavy metals by L-cysteine intercalated layered double hydroxide: Kinetic, isothermal and mechanistic studies, *J Colloid Interface Sci.* (2019). 10.1016/j.jcis.2019.12.028 31838351

[55] Xu, H. et al. [MoS_4_]^2-^ cluster bridges in Co-Fe layered double hydroxides for mercury uptake from S-Hg mixed flue gas. *Environ. Sci. Technol.* **51**, 10109–10116 (2017). 28759214 10.1021/acs.est.7b02537

[56] Hakami, O., Zhang, Y. & Banks, C. J. Thiol-functionalised mesoporous silica-coated magnetite nanoparticles for high efficiency removal and recovery of Hg from water. *Water Res.* **46**(2012), 3913–3922 (2012). 22608609 10.1016/j.watres.2012.04.032

[57] Kabiri, S., Tran, D. N., Azari, S. & Losic, D. Graphene-diatom silica aerogels for efficient removal of mercury ions from water. *ACS Appl. Mater. Interfaces* **7**(2015), 11815–11823 (2015). 25835089 10.1021/acsami.5b01159

[58] Khitous, M., Salem, Z. & Halliche, D. Sorption of Cr(VI) by MgAl-NO_3_ hydrotalcite in fixed-bed column: experiments and prediction of breakthrough curves. *Korean J. Chem. Eng.* **33**(2), 638–648. 10.1007/s11814-015-0170-3 (2015).

[59] Zhou, H., Jiang, Z., Wei, S. & Liang, J. Adsorption of Cd (II) from aqueous solutions by a novel layered double hydroxide FeMnMg-LDH. *Water Air Soil Pollut.* **229**, 1–16 (2018).

[60] Arafa, E. G., Mahmoud, R., Gadelhak, Y., & Gawad, O. F. A. Design, preparation, and performance of different adsorbents based on carboxymethyl chitosan/sodium alginate hydrogel beads for selective adsorption of Cadmium (II) and Chromium (III) metal ions. *Int. J. Biol. Macromol.* 132809 ‏ (2024). 10.1016/j.ijbiomac.2024.132809 38825296

[61] Awes, H. et al. Removal of Cu2+ metal ions from water using Mg-Fe layered double hydroxide and Mg-Fe LDH/5-(3-nitrophenyllazo)-6-aminouracil nanocomposite for enhancing adsorption properties. *Environ. Sci. Pollut. Res.* **28**(34), 47651–47667 (2021). 10.1007/s11356-021-13685-0 33895951

[62] Moaty, S. A., Farghali, A. A. & Khaled, R. Preparation, characterization and antimicrobial applications of Zn-Fe LDH against MRSA. *Mater. Sci. Eng. C Mater. Biol. Appl.* **68**, 184–193 (2016). 27524011 10.1016/j.msec.2016.05.110

[63] Qiao, C. et al. One-step synthesis of zincecobalt layered double hydroxide (ZneCo-LDH) nanosheets for high-efficiency oxygen evolution reaction. *J. Mater. Chem.* **3**, 6878e83 (2015).

[64] Ma, S. et al. Highly selective and efficient heavy metal capture with polysulfide intercalated layered double hydroxides. *J. Mater. Chem. A* **2**, 10280–10289 (2014).

[65] Zaher, A., Taha, M., Farghali, A. A. & Mahmoud, R. K. Zn/Fe LDH as a clay-like adsorbent for the removal of oxytetracycline from water: Combining experimental results and molecular simulations to understand the removal mechanism. *Environ. Sci. Pollut. Res.* **27**, 12256–12269 (2020). 10.1007/s11356-020-07750-3 31993907

[66] Soltani, R., Pelalak, R., Pishnamazi, M., Marjani, A. & Shirazian, S. A water-stable functionalized NiCo-LDH/MOF nanocomposite: Green synthesis, characterization, and its environmental application for heavy metals adsorption. *Arab. J. Chem.* **14**(4), 103052 (2021).

[67] An, H., Wang, Y., Wang, X., Li, N. & Zheng, L. The preparation of PANI/CA composite electrode material for supercapacitors and its electrochemical performance. *J. Solid State Electrochem.* **14**, 651–657 (2010).

[68] Shimizu, S. & Matubayasi, N. Surface area estimation: Replacing the Brunauer–Emmett–Teller model with the statistical thermodynamic fluctuation theory. *Langmuir* **38**, 7989–8002 (2022). 35715002 10.1021/acs.langmuir.2c00753 PMC9261182

[69] Zaitseva, E. S. & Tovbin, Y. K. Nnunoiform surfaces and the inflection point in polylayer adsorption isotherms. *Prot. Met. Phys. Chem. Surf.* **54**, 557–564 (2018).

[70] Zini. (2011). Chapter 16 - Abnormalities in leukocyte morphology and number, (Eds.): Anna, Porwit, Jeffrey, McCullough, Wendy, N., Erber. Blood and bone marrow pathology (Second edition), Churchill Livingstone, 247–261, ISBN: 9780702031472,10.1016/B978-0-7020-3147-2.00016-X*(*https://www.sciencedirect.com/science/article/pii/B978070203147200016X*).*

[71] Altomare, Craig Y. Y., & Kessler, M. Thrombocytosis: essential thrombocythemia and reactive causes,(Eds): Craig, S. Kitchens, Craig, M., Kessler, Barbara, A., Konkle, Michael, B., Streiff, David, A., Garcia, Consultative hemostasis & thrombosis (Fourth edition), Elsevier, 346–373, ISBN 9780323462020,10.1016/B978-0-323-46202-0.00019-4. (https://www.sciencedirect.com/science/article/pii/B9780323462020000194) (2019).

[72] Kobayashi, Y. The role of chemokines in neutrophil biology. *Front. Biosci.* **13**, 2400–7 (2008). 17981721 10.2741/2853

[73] Kolaczkowska, E. & Kubes, P. Neutrophil recruitment and function in health and inflammation. *Nat. Rev. Immunol.* **13**(3), 159–175 (2013). 23435331 10.1038/nri3399

[74] Balali-Mood M., Naseri K., Tahergorabi Z., Khazdair MR., & Sadeghi M. (2021). Toxic Mechanisms of Five Heavy Metals: Mercury, Lead, Chromium, Cadmium, and Arsenic JOURNAL=Frontiers in Pharmacology 12 *URL=* https://www.frontiersin.org/articles/10.3389/fphar.2021.643972OI=10.3389/fphar.2021.643972 *ISSN=1663-9812.* 10.3389/fphar.2021.643972 PMC8078867 33927623

[75] Clarke, E. G. C., & Clarke, M. L. Veterinary toxicology. Bailliere Tindall. (1975).

[76] Kura, A. U. et al. Acute oral toxicity and biodistribution study of zinc-aluminium-levodopa nanocomposite. *Nanoscale Res. Lett.* **10**, 1–11 (2015). 25852400 10.1186/s11671-015-0742-5 PMC4385219

[77] Figueiredo, M. P. et al. Iron-based layered double hydroxide implants: Potential drug delivery carriers with tissue biointegration promotion and blood microcirculation preservation. *ACS Omega* **3**(2018), 18263–18274 (2018).

[78] Medintz, I. L., Mattoussi, H. & Clapp, A. R. Potential clinical applications of quantum dots. *Int. J. Nanomed.* **3**, 151 (2008). 10.2147/ijn.s614 PMC2527673 18686776

[79] Debbage, P. Targeted drugs and nanomedicine: Present and future. *Curr. Pharm. Des.* **15**(2), 153–172 (2009) (**2009**). 19149610 10.2174/138161209787002870

[80] Scheurich, D. & Woeltje, K. Skin and soft tissue infections due to CA-MRSA. *Mo. Med.* **106**, 274–276 (2009). 19753919

[81] Behrens, A. I. V., Pena, M. J., Alonso, T. & Kissel,. Comparative uptake studies of bioadhesive and non-bioadhesive nanoparticles in human intestinal cell lines and rats: the effect of mucus on particle adsorption and transport. *Pharm. Res.* **19**, 1185–1193 (2002). 12240945 10.1023/a:1019854327540

[82] Islam, T. & Harisinghani, M. G. Overview of nanoparticle use in cancer imaging. *Cancer Biomark.* **5**, 61–67 (2009). 19414922 10.3233/CBM-2009-0578 PMC12922814

[83] Lai, S. K., Wang, Y. Y. & Hanes, J. Mucus-penetrating nanoparticles for drug and gene delivery to mucosal tissues. *Adv. Drug Deliv. Rev.* **61**, 158–171 (2009). 19133304 10.1016/j.addr.2008.11.002 PMC2667119

[84] Jin, Y., Hea-Eun, C. & Soo-Jin, C. Acute Oral toxicity and kinetic behaviors of inorganic layered nanoparticles. *J. Nanomater.*10.1155/2013/628381 (2013).

[85] Govea-Alonso, D. O. et al. Assessing the adjuvant effect of layered double hydroxides (LDH) on BALB/c rat. *Mater.* **2023**(16), 5467 (2023). 10.3390/ma16155467 PMC10419364 37570172

[86] Manda Damasceno Leão, da Silva J. R., Agostini J. F., Dal Santo G., Vieira L. D., Neto J. D. C. S., Ramos K. R. D. L. P., da Silva T. G., Alvarez-Lorenzo C., Wanderley A. G., & Soares-Sobrinho J. L., Efficacy and safety of nanoparticles of glibenclamide and organomodified layered double hydroxides in diabetics 10.1016/j.ijpharm.2023.122678 (2023). 10.1016/j.ijpharm.2023.122678 36738803

[87] De Araújo, M. A. et al. Layered double hydroxides (LDHs) as efficient and safe carriers for miRNA inhibitors: In vitro and in vivo assessment of biocompatibility. *Chem. Biol. Interact.* **391**, 110874. 10.1016/j.cbi.2024.110874 (2024). 38311162 10.1016/j.cbi.2024.110874

[88] Betersmann, D. & Hartwig, A. Carcinogenic metal compounds: Recent insight into molecular and cellular mechanisms. *Arch. Toxicol.* **82**(8), 493–512. 10.1007/s00204-0080313-y (2008). 18496671 10.1007/s00204-008-0313-y

