# Supplementary material for: Environmental and sustainable valorization of spent adsorbent: safety and acute toxicity evaluation in rats via probit analysis
Source: Sci Rep. 2026 May 18;16:15333. doi: 10.1038/s41598-026-50808-4 (PMC13183944; doi:10.1038/s41598-026-50808-4)
Supplement: Supplementary file 1 — Supplementary Information. [file 41598_2026_50808_MOESM1_ESM.pdf]

Institutional Animal Care and Use Committee
Beni-Suef University (BSU-IACUC)

Approval Number

Dear Dr.

This letter is to inform you that the following animal protocol was approved by BSU-IACUC reviewers

Approval number:- 022-459

Principal Investigator:- Saher Atia Abdelkalik

Approval date:- 22/10/2023

Faculty:- faculty of postgraduate studies for advanced sciences

Title:- Recycling approach:improving the quality of industrial wastewater by using layered double hydroxide (LDH).

Chair BSU-IACUC

Contact

http://www.bsu.edu.eg/IACUC.aspx

١١/١ ٢٠٢٣
